# The association between fasting plasma glucose variability and incident eGFR decline: evidence from two cohort studies

**DOI:** 10.1186/s12889-023-15463-8

**Published:** 2023-03-27

**Authors:** Niloofar Deravi, Yasaman Sharifi, Fatemeh Koohi, Seyed Saeed Tamehri Zadeh, Soroush Masrouri, Fereidoun Azizi, Farzad Hadaegh

**Affiliations:** 1 Prevention of Metabolic Disorders Research Center, Research Institute for Endocrine Sciences, Shahid Beheshti University of Medical Sciences, No. 24, Parvaneh Street, Velenjak, Tehran, 19395-4763 Iran; 2 Student Research Committee, School of Medicine, Shahid Beheshti University of Medical Sciences, Tehran, Iran; 3 Department of Radiology, School of Medicine, Iran University of Medical Sciences, Tehran, Iran; 4 Endocrine Research Center, Research Institute for Endocrine Sciences, Shahid Beheshti University of Medical Sciences, Tehran, Iran

**Keywords:** Glycemic variability, Fasting plasma glucose, Type 2 diabetes, Estimated glomerular filtration rate decline, eGFR decline

## Abstract

**Background:**

Glycemic variability (GV) is developing as a marker of glycemic control, which can be utilized as a promising predictor of complications. To determine whether long-term GV is associated with incident eGFR decline in two cohorts of Tehran Lipid and Glucose Study (TLGS) and Multi-Ethnic Study of Atherosclerosis (MESA) during a median follow-up of 12.2 years.

**Methods:**

Study participants included 4422 Iranian adults (including 528 patients with T2D) aged ≥ 20 years from TLGS and 4290 American adults (including 521 patients with T2D) aged ≥ 45 years from MESA. The Multivariate Cox proportional hazard models were used to assess the risk of incident eGFR decline for each of the fasting plasma glucose (FPG) variability measures including standard deviation (SD), coefficient of variation (CV), average real variability (ARV), and variability independent of the mean (VIM) both as continuous and categorical variables. The time of start for eGFR decline and FPG variability assessment was the same, but the event cases were excluded during the exposure period.

**Results:**

In TLGS participants without T2D, for each unit change in FPG variability measures, the hazards (HRs) and 95% confidence intervals (CI) for eGFR decline ≥ 40% of SD, CV, and VIM were 1.07(1.01–1.13), 1.06(1.01–1.11), and 1.07(1.01–1.13), respectively. Moreover, the third tertile of FPG-SD and FPG-VIM parameters was significantly associated with a 60 and 69% higher risk for eGFR decline ≥ 40%, respectively. In MESA participants with T2D, each unit change in FPG variability measures was significantly associated with a higher risk for eGFR decline ≥ 40%.Regarding eGFR decline ≥ 30% as the outcome, in the TLGS, regardless of diabetes status, no association was shown between FPG variability measures and risk of eGFR decline in any of the models; however, in the MESA the results were in line with those of GFR decline ≥ 40%.Using pooled data from the two cohorts we found that generally FPG variability were associated with higher risk of eGFR decline ≥ 40% only among non-T2D individuals.

**Conclusions:**

Higher FPG variability was associated with an increased risk of eGFR decline in the diabetic American population; however, this unfavorable impact was found only among the non-diabetic Iranian population.

**Supplementary Information:**

The online version contains supplementary material available at 10.1186/s12889-023-15463-8.

## Introduction

Glycemic variability (GV), also known as blood glucose swings, includes a wide range of blood glucose variations that occur throughout the day, including hypoglycemic periods and postprandial spikes, as well as fluctuations that occur at the same time on different days. Diabetes complications are associated with GV in both type one diabetes and type 2 diabetes (T2D) patients [1–5]. Accordingly, GV was shown to be mostly associated with oxidative stress [6] and an increased incidence of hypoglycemia, a trigger of inflammatory processes [5, 7]. GV is measured on a short- and long-term basis [5]. Evidence supports the importance of this index both as a criterion for glycemic control and in the prevention of complications among patients with diabetes [1, 2, 4, 5, 8–12].

Short-term GV refers to between-days or within-day glycemic fluctuations, which are often measured via continuous glucose monitoring mostly in patients with type 1 diabetes [13]. Long-term GV refers to glycemic fluctuations over months to years, mostly measured by visit-to-visit variability in either fasting plasma glucose (FPG) or HbA1c in patients with diabetes [13]. Previous studies have shown that long-term GV may predict chronic kidney disease (CKD), the main microvascular complication of diabetes leading to both morbidity and mortality [14–18] with a high prevalence and incidence rate among both Iranian and American populations [19–22].The Food and Drug Administration (FDA) committee and National Kidney Foundation (NKF) have published a series of studies to investigate whether eGFR declines less than 50% could be defined as an important kidney endpoint [23–26]. The committee reported that a 30% decline in eGFR can be regarded as a reliable surrogate endpoint in some circumstances; however, a 40% eGFR decline could present stronger evidence [27–29]. Accordingly, a meta-analysis of 1.7 million participants reported the average adjusted 10-year risk of end-stage renal disease (ESRD) for eGFR declines of 40% and 30%, were 83% and 64%, respectively [24].

Previous studies mainly conducted among T2D patients in the East Asian region have investigated the effect of HbA1c variation on eGFR decline [30–32], however, there is a paucity of information on the effect of FPG variability on eGFR decline in individuals with T2D [32]. Therefore, the current study for the first time investigated the association of long-term FPG variability with eGFR decline ≥ 30 and 40% in non-CKD adults with and without T2D in the Tehran Lipid and Glucose Study (TLGS), as well as in the participants of the Multi-Ethnic Study of Atherosclerosis (MESA) study during about one decade of follow-up.

## Methods

### Study population

Study participants were selected from participants of TLGS, an ongoing large-scale population-based cohort study conducted on a representative population of Tehran city, the capital of Iran. TLGS aims to determine the risk factors for non-communicable diseases. The design of TLGS has been published before [33, 34]. In brief, in phase 1 (1999–2001) 15,005 participants ≥ 3 years entered the study; the data collection has been continued since then at approximately three-year intervals in follow-up phases (i.e., phases 2 (2002–2005), 3 (2005–2008), 4 (2008–2011), 5 (2011–2014), and 6 (2014–2017)). Moreover, 3555 participants entered the cohort in phase 2 of the study and were subsequently followed in phases 3, 4, 5, and 6.

**Fig. 1:**
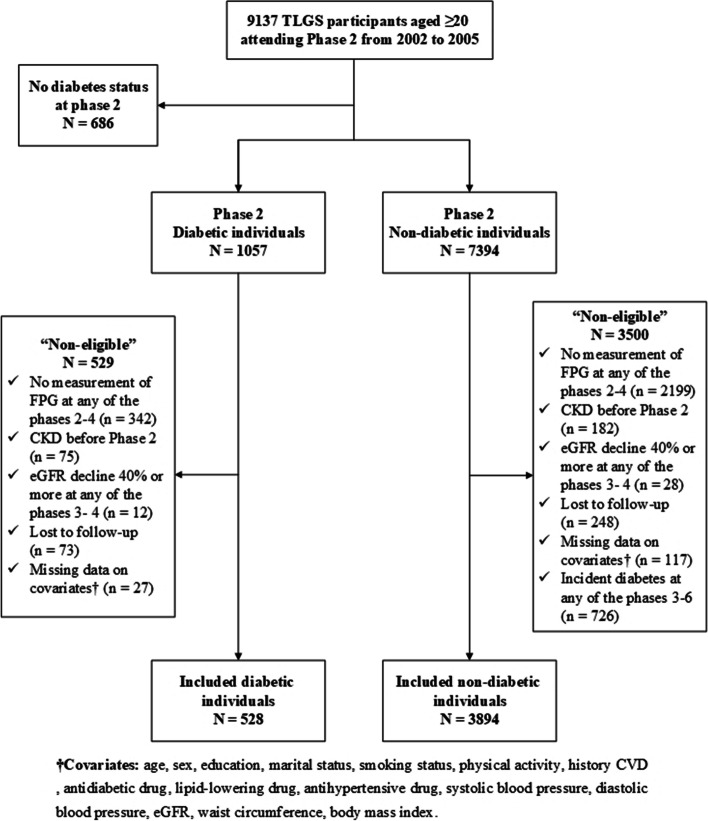
TLGS study participants’ flowchart

In the present study, we included 9137 participants of the TLGS cohort (1057 patients with T2D) aged ≥ 20 years who participated in phase 2 (as the baseline phase). We aimed to calculate the visit-to-visit variability (VVV) of FPG; therefore, we included those with available FPG values in phases 3, and 4.. Consequently, we considered 2002–2011 as the exposure period. We excluded individuals with no measurement of FPG at any of the phases 2–4, diagnosed CKD before phase 2 and eGFR decline ≥ 40% during the measurement period (i.e., at any of the phases 3–4), lost to follow-up at any of the phases, and missing data on covariates. Among non-diabetes group, those with incident T2D at any of the phases 3–6 were also excluded. Finally, 4422 participants (including 528 patients with T2D) remained for data analysis and followed till March 2018. Using a similar approach, for the eGFR decline ≥ 30%, 4,181 individuals (483 patients with T2D) were entered in the data analysis. Figure 1 demonstrates the flowchart of the TLGS study participants.

**Fig. 2:**
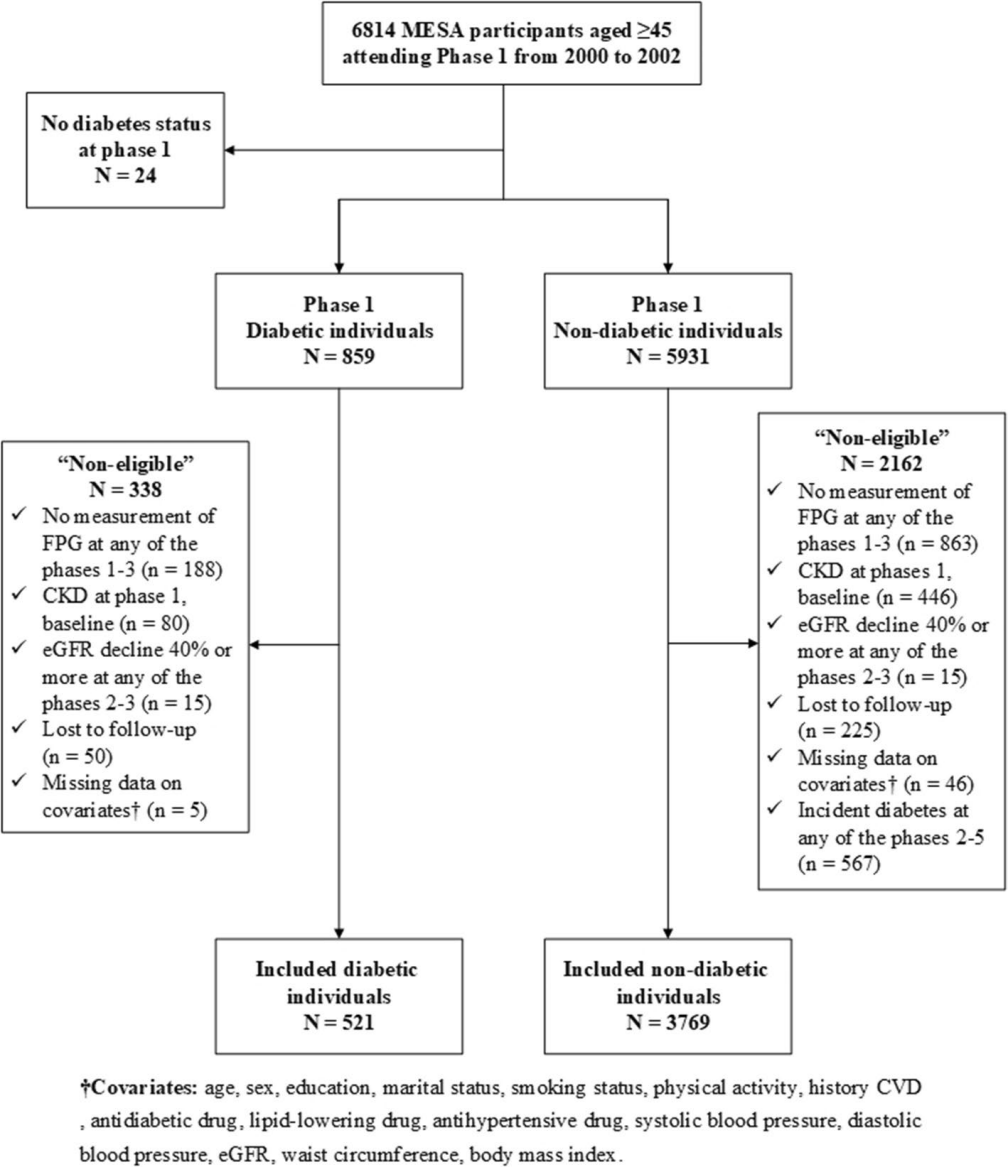
MESA study participants’ flowchart

The MESA dataset, from a longitudinal study, was also used. Participants aged 45 to 84 years at baseline, from six sites around the United States, were oversampled by four ethnic/racial groups. Design and objectives have been described in detail elsewhere [35]. Briefly, after the baseline examination, there have been five additional follow-up visits at biennial intervals, the most recent ongoing in 2016–18. Institutional Review Board approval was granted at each site and informed consent was obtained from each participant. MESA included 6814 participants (859 patients with T2D aged ≥ 45 years who participated in phase 1 (Sep 2002 to Feb 2004). Similar to the process for the TLGS cohort we measured the VVV of FPG in MESA participants; therefore, we included those with available FPG values in sequential phases 1, 2 (Mar 2004 to Sep 2005), and 3 (2005 to May 2007). We excluded individuals with no measurement of FPG at any of the phases 1–3, diagnosed CKD at phases 1, eGFR decline ≥ 40% at any of the phases 2–3, lost follow-up at any of the phases, and missing data on covariates. Those with incident T2D at any of the phases 2–5 in the non-diabetic group were also excluded. Finally, 4290 participants (including 521 patients with T2D) remained for data analysis. Hence, the final samples were followed for incident eGFR decline ≥ 40% after the measurement period. Using the similar approach, for the eGFR decline ≥ 30%, 4,290 individuals (521 patients with T2D) were entered in the data analysis. Figure 2 demonstrates the flowchart of the MESA study participants.

### Clinical and laboratory measurements

We collected the baseline characteristics including data on age, sex, drug, family, past medical history, and smoking status (never or ever) through a standard questionnaire and measured body weight and height, diastolic blood (DBP), and systolic (SBP) pressure during a clinical examination. We calculated body mass index (BMI) as weight (kg) divided by the square of height (m^2^). Using a standard mercury sphygmomanometer, we measured blood pressure twice in a seating position after 15 min of rest. We also drew blood samples for the measurement of FPG, total cholesterol (TC), and creatinine levels, after 12–14 h of overnight fasting, from all participants, assayed serum creatinine levels by the Jaffe kinetic calorimetric method, and analyzed the samples once internal quality control met the standard satisfactory criteria. The standard 2-h post-load plasma glucose (2-HPG) test was also performed for those not on glucose lowering drugs. Physical activity level was assessed using the Modifiable Activity Questionnaire (Low, moderate-high) [36]. Moderate-high Physical activity level was defined as achieving a score ≤ 600 MET-minutes per week [37]. Details of laboratory assessments were reported previously [38]. Detailed description of MESA clinical and laboratory measurements has been published elsewhere [35].

We defined VVV as an intra individual variability in FPG levels recorded across three consecutive examinations in both TLGS and MESA. We used four indices of variability: (a) coefficient of variation (CV), (b) Standard deviation (SD), (c) average real variability (ARV), and (d) variability independent of the mean (VIM). We calculated VIM as 100 * SD/mean^β^, where β is the regression coefficient based on the natural logarithm of SD on the natural logarithm of the mean. We calculated ARV according to the following formula, where n denotes the number of measures of FPG [39].

$$\mathrm{ARV}=\frac{1}{n-1}{\sum }_{i=1}^{n-1}\left|Value(i+1\right)-Value(i)|$$

ARV is the average of absolute differences between consecutive values.

### Definition of outcomes and variables

The main outcome for this study was a reduction in eGFR of ≥ 40 and ≥ 30% from the baseline. eGFR was estimated by use of the Chronic Kidney Disease Epidemiology Collaboration (CKD-EPI) equation.

141*min (Scr/ƙ, 1)α *max (Scr/ƙ, 1)-1.209 *0.993age *1.018 (if female)*1.159 (if black).

In this equation, we measured serum creatinine (Scr) in mg/dL, and age in years. ƙ is 0.9 and 0.7 for women and men, respectively; α is -0.329 and -0.411 for women and men; min indicates the minimum of Scr/ƙ or 1, and max indicates a maximum of Scr/ƙ or 1. eGFR was expressed as mL/min/1.73 m2.

The percentage of change in eGFR was calculated as:

$$\frac{\text{Follow up measurement}-\text{ Baseline measurement}}{\text{Baseline measurement}} \times 100$$

We defined T2D as FPG of ≥ 126 mg/dl or current use of antidiabetic drugs. Moreover, prevalent cardiovascular disease (CVD) was a self-reported CVD history with a prior diagnosis of CVD by a physician. Smoking was categorized as ever smoker (current or past) versus non-smoker. Smoking was categorized as ever smoker (current or past) versus non-smoker. Hypertension, was defined as the presence of at least one of the following criteria: (a) having SBP ≥ 140 mm Hg, (b) having DBP ≥ 90 mm Hg, and (c) initiation of anti-hypertensive drugs usage.

### Statistical methods

We showed the baseline characteristics of participants as frequency (%) for categorical variables and the mean ± standard deviation (SD) for continuous variables. We also categorized participants with and without T2D into 3 groups according to the tertiles of FPG-SD and compared the baseline characteristics across these tertiles. Chi-square test and analysis of variance were used to compare the clinical and demographic characteristics, as appropriate.

The follow-up time used for time-to-event analyses was defined as the time from baseline date (phase 2) to either eGFR decline ≥ 40 or 30% in phases 5 or 6, or date of last data collection or death, whichever occurred first. Only the first occurrence of each outcome was used for analysis.

Each glucose variability measure was categorized by tertiles with a reference level of the lowest tertile. For individuals with incident eGFR decline, survival time was defined as the mid-time between the entered date and the event date. Linear trends across the tertiles were calculated by including the tertile as a continuous variable in the models. Moreover, to estimate adjusted HRs and 95% CIs for incident eGFR decline associated with GV, we applied multivariable Cox proportional hazard (Cox PH) models, separately for participants with and without T2D. We used the measures of FPG variability including CV, SD, ARV, and VIM as a continuous variable (for each unit change) and tertiles in Cox PH models (the lowest tertile was considered as the reference). We also created four models in each dataset and adjusted for well known risk factors for CKD that were reported in a systematic review in this field. [40, 41]: Model 1: adjusted for age and sex at baseline (phase 2). Model 2: Model 1 + marital status, education, ever smoking, prevalent CVD, physical activity, anti-diabetic drug use, anti-hypertensive drug, lipid-lowering drug, BMI, WC, SBP, DBP, eGFR, and FPG at baseline (phase 2). Model 3: Model 1 + marital status, education, ever smoking, prevalent CVD, and physical activity at baseline (phase 2), and anti-diabetic drug use, anti-hypertensive drug, and lipid-lowering drug over phases 2–4, and average BMI, WC, SBP, DBP, and eGFR over phases 2. And Model 4: Model 3 + the average FPG during the measurement period. We conducted the above-mentioned analysis in the pooled data of TLGS and MESA cohorts using stratified Cox regression analysis and R version 3.6.2. Only, in the TLGS cohort, the above analysis was performed for 2-HPG among non-type 2 diabetic population; the association between this parameter with eGFR decline among newly diagnosed type 2 diabetic population was also examined only in Model 1. We calculated the median follow-up between 2002 (baseline phase) and 2018 (the end of the study) and assessed the PH assumptions in Cox models with the Schoenfeld residuals test and log–log plots, showing all proportional assumptions were appropriate. We performed the statistical analyses using STATA version 14. We also considered a *P*-value of < 0.05 as statistically significant.

## Results

### The TLGS cohort

Participants included 528 patients with T2D (women = 326) with a mean (SD) age of 52.5 (10.6) years and 3894 participants without T2D (women = 2288) with a mean (SD) age of 40.5 (13.1) years. Baseline characteristics of the study population for participants with and without T2D across tertiles of FPG-SD are presented in Table 1. Generally, compared to the first tertile of FPG-SD, subjects with T2D at the third tertile had higher lipid-lowering drug use as well as higher FPG levels at baseline, phases 3, and 4. There was also a significant difference between ages of participants between teriles of FPG-SD. Moreover, among participants without T2D, those in the third tertile of FPG-SD generally had higher BMI, WC, and FPG levels at phases 3 and 4 compared to the reference group. There was also a significant difference between the levels of education of participants between teriles of FPG-SD.

**Table 1:** Baseline characteristics of participants across tertiles of SD for fasting plasma glucose in TLGS, Tehran Lipid and Glucose Study (2002–2018)

Characteristics	**With diabetes (** ***n*** ** = 528)**	**T1 (** ***n*** ** = 176)**	**T2 (** ***n *** **= 176)**	**T3 (** ***n*** ** = 176)**	***P*** ** value**
Age (year)	**52.51 ± 10.64**	**52.37 ± 11.62**	**53.99 ± 9.84**	**51.18 ± 10.26**	**0.044**
Sex (women)	326 (61.74)	112 (63.64)	97 (55.11)	117 (66.48)	0.074
Marital status (married)	457 (86.55)	144 (81.82)	156 (88.64)	157 (89.20)	0.078
Education (high school and more)	124 (23.48)	42 (23.86)	42 (23.86)	40 (22.73)	0.959
Antihypertensive drug	124 (23.48)	48 (27.27)	37 (21.02)	39 (22.16)	0.338
Lipid-lowering drug	**81 (15.34)**	**18 (10.23)**	**25 (14.20)**	**38 (21.59)**	**0.011**
Current smoking	109 (20.64)	34 (19.32)	43 (24.43)	32 (18.18)	0.304
Moderate-high physical activity	315 (59.66)	103 (58.52)	101 (57.39)	111 (63.07)	0.516
BMI (Kg/m^2^)	29.43 ± 4.75	29.61 ± 4.89	29.02 ± 4.30	29.65 ± 5.04	0.386
WC (cm)	98.87 ± 10.85	99.09 ± 11.22	98.67 ± 9.34	98.84 ± 11.39	0.937
SBP (mmHg)	127.91 ± 20.12	126.87 ± 19.61	129.55 ± 19.78	127.31 ± 20.96	0.408
DBP (mmHg)	78.35 ± 10.19	78.35 ± 10.19	79.87 ± 11.02	78.77 ± 10.50	0.380
eGFR (mL/min/1.73 m2)	88.67 ± 15.11	89.01 ± 16.06	87.82 ± 14.63	89.17 ± 14.63	0.659
FPG (mg/dl)	**156.45 ± 53.00**	**132.38 ± 36.75**	**154.43 ± 40.29**	**182.55 ± 64.92**	** < 0.001**
FPG at phase 3 (mg/dl)	**130.66 ± 54.9**	**130.66 ± 37.70**	**150.39 ± 38.41**	**188.64 ± 68.55**	** < 0.001**
FPG at phase 4 (mg/dl)	**166.2 ± 60.6**	**135.39 ± 36.81**	**163.13 ± 42.28**	**198.53 ± 76.18**	** < 0.001**
Anti-diabetic drug at baseline	**231 (43.75)**	**60 (34.09)**	**75 (42.61)**	**96 (54.55)**	**0.001**
	Without diabetes (*n* = 3,894)	T1 (*n* = 1,331)	T2 (*n* = 1,331)	T3 (*n* = 1,331)	*P* value
Age (year)	40.49 ± 13.09	40.18 ± 13.04	40.28 ± 13.02	41.02 ± 13.21	0.201
Sex (women)	2,288 (58.76)	808 (60.71)	740 (58.13)	740 (57.36)	0.190
Marital status (married)	3,144 (80.74)	1,056 (79.34)	1,027 (80.68)	1,061 (82.25)	0.168
Education (high school and more)	**1,318 (33.85)**	**436 (32.76)**	**466 (36.61)**	**416 (32.25)**	**0.039**
Antihypertensive drug	220 (5.65)	79 (5.94)	66 (5.18)	75 (5.81)	0.675
Lipid-lowering drug	113 (2.90)	40 (3.01)	26 (2.04)	47 (3.64)	0.052
Current smoking	812 (20.85)	267 (20.06)	258 (20.27)	787 (61.01)	0.318
Moderate-high physical activity	2458 (63.12)	858 (64.46)	813 (63.86)	503 (38.99)	0.149
BMI (Kg/m^2^)	**26.82 ± 4.44**	**26.64 ± 4.44**	**26.76 ± 4.39**	**27.08 ± 4.49**	**0.033**
WC (cm)	**89.22 ± 11.70**	**88.34 ± 11.72**	**89.19 ± 11.40**	**90.15 ± 11.91**	** < 0.001**
SBP (mmHg)	113.15 ± 16.00	112.36 ± 15.48	113.34 ± 16.16	113.79 ± 16.34	0.066
DBP (mmHg)	73.54 ± 10.07	73.11 ± 9.87	73.55 ± 10.25	73.98 ± 10.08	0.085
eGFR (mL/min/1.73 m2)	96.59 ± 16.57	96.77 ± 16.51	96.64 ± 16.22	96.37 ± 16.98	0.818
FPG (mg/dl)	88.44 ± 8.04	88.33 ± 6.75	88.63 ± 7.71	88.37 ± 9.47	0.579
FPG at phase 3 (mg/dl)	**87.66 ± 7.74**	**88.11 ± 6.64**	**87.45 ± 7.48**	**87.41 ± 8.93**	**0.033**
FPG at phase 4 (mg/dl)	**93,20 ± 8.10**	**89.66 ± 6.51**	**92.53 ± 6.86**	**97.50 ± 8.73**	** < 0.001**

Data are represented as mean ± standard deviation for continuous variables and frequency (percent) for categorical variables

*T* tertile, *CVD* cardiovascular disease, *BMI* body mass index, *WC* waist circumference, *SBP* systolic blood pressure, *DBP* diastolic blood pressure, *eGFR* estimated glomerular filtration rate; FPG Fasting plasma glucose

After a median follow-up of 12.2 years (interquartile range: 11.1–13.3 years), 131 incident eGFR decline ≥ 30% and 72 incident eGFR decline ≥ 40% among subjects with T2D occurred; the corresponding values for non-diabetic participants were, 629 and 115, respectively.

Table 2 shows the association of FPG variability as a continuous variable in four models with an eGFR decline ≥ 40%. Among participants without T2D, each unit change in FPG variability measures was significantly associated with a higher risk for eGFR decline ≥ 40% in all models of FPG-SD, FPG-CV, and FPG-VIM; the corresponding HRs and 95% CIs in the last models were 1.07 (1.01–1.13), 1.06 (1.01–1.11), and 1.07 (1.01–1.13), respectively. However, among participants with T2D, none of these measures were associated with eGFR decline ≥ 40% events, even in model 1.

**Table 2:** HRs and 95% CIs of incident eGFR decline ≥ 40% according to each unit increase in FPG variability measures in Tehran Lipid and Glucose Study

Variability measures	Model 1		Model 2		Model 3		Model 4	
	HR (95% CI)	*P* value	HR (95% CI)	*P* value	HR (95% CI)	*P* value	HR (95% CI)	*P* value
SD								
With diabetes	1.00(0.99-1.01)	0.645	0.99 (0.98-1.00)	0.167	0.99 (0.99-1.00)	0.258	0.99 (0.98-1.00)	0.106
Without diabetes	1.08(1.02-1.13)	**0.006**	1.06(1.01-1.12)	**0.020**	1.07 (1.01-1.13)	**0.012**	1.07 (1.01-1.13)	**0.014**
CV								
With diabetes	0.99 (0.98-1.01)	0.511	0.99 (0.97-1.01)	0.158	0.99 (0.97-1.01)	0.190	0.99 (0.97-1.00)	0.138
Without diabetes	1.06(1.01-1.12)	**0.012**	1.05 (1.00-1.11)	**0.033**	1.06 (1.01-1.11)	**0.017**	1.06 (1.01-1.11)	**0.017**
ARV								
With diabetes	1.00(0.99-1.01)	0.874	1.00 (0.99-1.00)	0.310	1.00 (0.99-1.00)	0.513	1.00 (0.99-1.00)	0.309
Without diabetes	1.03(0.99-1.07)	0.158	1.03(0.99-1.07)	0.137	1.03 (0.99-1.07)	0.183	1.03 (0.99-1.07)	0.208
VIM								
With diabetes	1.00 (0.98-1.01)	0.463	0.99 (0.98-1,00)	0.252	0.99 (0.98-1.00)	0.233	0.99 (0.98-1.00)	0.250
Without diabetes	1.07(1.01-1.13)	**0.016**	1.06 (1.00-1.11)	**0.041**	1.07 (1.01-1.13)	**0.019**	1.07 (1.01-1.13)	**0.018**

Model 1: adjusted for age and sex at baseline (phase 2)

Model 2: Model 1 + marital status, education, ever smoking, prevalent CVD, physical activity, anti-diabetic drug use, anti-hypertensive drug, lipid-lowering drug, BMI, WC, SBP, DBP, eGFR, and FPG at baseline (phase 2)

Model 3: Model 1 + marital status, education, ever smoking, prevalent CVD, and physical activity at baseline (phase 2), and anti-diabetic drug use, anti-hypertensive drug, and lipid-lowering drug over phases 2–4, and average BMI, WC, SBP, DBP, and eGFR over phases 2–4

Model 4: Model 3 + average FPG

The associations of FPG variability as a categorical variable with incident eGFR decline ≥ 40% are presented in Table 3. Generally, for SD and VIM in all of the models, a significant increasing trend was observed for incident eGFR decline among participants without T2D. Furthermore, among participants without T2D, the third tertiles of FPG-SD, and FPG-VIM were associated with higher risks in all models (the corresponding HRs and 95% CIs in the last model were 1.60 (1.01–2.54), and 1.69(1.06–2.69), respectively). Whereas, no trends for incident eGFR decline were observed in participants with T2D in any of the FPG variability measures.

**Table 3:** HRs and 95% CIs of incident eGFR decline ≥ 40% according to tertiles of FPG variability measures in Tehran Lipid and Glucose Study

Variability measures	**Model 1**	**Model 2**	**Model 3**	**Model 4**
	HR (95% CI)	HR (95% CI)	HR (95% CI)	HR (95% CI)
**With diabetes**				
SD				
T1	1.00 (reference)	1.00 (reference)	1.00 (reference)	1.00 (reference)
T2	1.20 (0.69–2.10)	1.06 (0.59–1.90)	0.93 (0.53–1.66)	0.88 (0.49–1.58)
T3	0.98 (0.54–1.77)	0.76 (0.39–1.47)	0.77 (0.42–1.44)	0.67 (0.33–1.33)
P _trend_	0.968	0.414	0.417	0.254
CV				
T1	1.00 (reference)	1.00 (reference)	1.00 (reference)	1.00 (reference)
T2	1.04 (0.60–1.82)	0.96 (0.53–1.73)	0.80 (0.44–1.43)	0.77 (0.42–1.39)
T3	0.94 (0.53–1.66)	0.74 (0.40–1.37)	0.74 (0.41–0.34)	0.70 (0.38–1.29)
P _trend_	0.828	0.332	0.327	0.253
ARV				
T1	1.00 (reference)	1.00 (reference)	1.00 (reference)	1.00 (reference)
T2	1.47 (0.84–2.58)	1.29 (0.71–2.33)	0.99 (0.55–1.79)	0.94 (0.52–1.72)
T3	1.09 (0.60–2.01)	0.84 (0.43–1.65)	0.83 (0.44–1.55)	0.71 (0.35–1.44)
P _trend_	0.749	0.588	0.539	0.344
VIM				
T1	1.00 (reference)	1.00 (reference)	1.00 (reference)	1.00 (reference)
T2	1.10 (0.63–1.92)	1.18 (0.67–2.09)	1.09 (0.62–1.92)	1.10 (0.62–1.93)
T3	0.94 (0.53–1.66)	0.84 (0.46–1.51)	0.82 (0.46–1.47)	0.83 (0.46–1.49)
P _trend_	0.835	0.577	0.512	0.746
**Without diabetes**				
SD				
T1	1.00 (reference)	1.00 (reference)	1.00 (reference)	1.00 (reference)
T2	1.28 (0.80–2.07)	1.37 (0.84–2.21)	1.32 (0.81–2.13)	1.31 (0.81–2.12)
T3	**1.61 (1.02–2.53)**	**1.67 (1.05–2.64)**	**1.61 (1.02–2.55)**	**1.60 (1.01–2.54)**
P _trend_	**0.039**	**0.029**	**0.041**	**0.047**
CV				
T1	1.00 (reference)	1.00 (reference)	1.00 (reference)	1.00 (reference)
T2	1.36 (0.85–2.18)	1.43 (0.89–2.30)	1.43 (0.90–2.31)	1.44 (0.89–2.30)
T3	1.53 (0.97–2.42)	1.56 (0.98–2.48)	1.55 (0.98–2.47)	1.55 (0.97–2.46)
P _trend_	0.071	0.064	0.065	0.068
ARV				
T1	1.00 (reference)	1.00 (reference)	1.00 (reference)	1.00 (reference)
T2	1.08 (0.68–1.70)	1.18 (0.74–1.88)	1.21 (0.76–1.93)	1.21 (0.76–1.92)
T3	1.27 (0.82–1.96)	1.38 (0.89–2.15)	1.30 (0.84–2.01)	1.28 (0.82–1.99)
P _trend_	0.286	0.147	0.242	0.272
VIM				
T1	1.00 (reference)	1.00 (reference)	1.00 (reference)	1.00 (reference)
T2	1.42 (0.88–2.28)	1.49 (0.93–2.41)	1.49 (0.93- 2.39)	1.49 (0.92–2.40)
T3	**1.65 (1.04–2.62)**	**1.66 (1.04–2.67)**	**1.69 (1.06–2.69)**	**1.69(1.06–2.69)**
P _trend_	**0.034**	**0.035**	**0.029**	**0.029**

Model 1: adjusted for age and sex at baseline (phase 2)

Model 2: Model 1 + marital status, education, ever smoking, prevalent CVD, physical activity, anti-diabetic drug use, anti-hypertensive drug, lipid-lowering drug, BMI, WC, SBP, DBP, eGFR, and FPG at baseline (phase 2)

Model 3: Model 1 + marital status, education, ever smoking, prevalent CVD, and physical activity at baseline (phase 2), and anti-diabetic drug use, anti-hypertensive drug, and lipid-lowering drug over phases 2–4, and average BMI, WC, SBP, DBP, and eGFR over phases 2–4

Model 4: Model 3 + average FPG

Supplementary Tables 1 and 2 show the association of FPG variability both as a continuous and categorical variable in four models with an eGFR decline ≥ 30%. Regardless of diabetes status, no association was shown between FPG variability measures and risk of eGFR decline in any of the models.

As a sensitivity analysis, we examined the association of 2-HPG variability both as continuous and categorical variables with eGFR declines ≥ 30% and 40% among participants without T2D and newly diagnosed T2D not on glucose lowering medications (Supplementary Tables 3, 4, 5, 6, and 7, respectively). No association was shown between 2-HPG variability measures and risk of eGFR decline in any of the models in both non-diabetic individuals and newly diagnosed T2D participants.

### MESA cohort

A population of 521 participants (women = 236) with T2D with a mean (SD) age of 63.2 (9.1) years and 3769 participants without T2D (women = 1968) with a mean (SD) age of 60.4 (9.9) years were evaluated. Baseline characteristics of the study population for participants with and without T2D across tertiles of FPG-SD are presented in Table 4. Compared to the first tertile of FPG-SD, subjects with T2D at the third tertile were generally younger, and had higher BMI as well as FPG levels at baseline, phases 2, and 3. Furthermore, among participants without T2D, those in the third tertile of FPG-SD (compared to the first tertile) were generally younger and had higher BMI, WC, and FPG levels in phases 3, and 4. There was also a significant difference between smoking status of participants between teriles of FPG-SD.

**Table 4:** Baseline characteristics of the participants across tertiles of SD for fasting plasma glucose in Multi-Ethnic Study of Atherosclerosis study

Characteristics	**With diabetes (** ***n *** **= 521)**	**T1 (** ***n*** ** = 176)**	**T2 (** ***n *** **= 172)**	**T3 (** ***n*** ** = 173)**	***P*** ** value**
Age (year)	63.21 ± 9.13	64.85 ± 8.82	64.67 ± 8.85	60.10 ± 8.96	** < 0.001**
Sex (women)	236 (45.30)	83 (47.16)	72 (41.86)	81 (46.82)	0.541
Marital status (married)	320 (61.42)	108 (61.36)	110 (63.95)	102 (58.96)	0.635
Education (high school and more)	281 (53.93)	105 (59.66)	86 (50.00)	90 (52.02)	0.161
Antihypertensive drug	317 (60.84)	106 (60.23)	114 (66.28)	97 (56.07)	0.148
Lipid-lowering drug	142 (27.26)	47 (26.70)	47 (27.33)	48 (27.75)	0.976
Current smoking	259 (49.71)	82 (46.59)	88 (51.16)	89 (51.45)	0.595
Moderate-high physical activity	279 (53.55)	96 (54.55)	91 (52.51)	92 (53.18)	0.947
BMI (Kg/m^2^)	**30.51 ± 5.63**	**29.59 ± 5.18**	**30.88 ± 6.05**	**31.08 ± 5.58**	**0.028**
WC (cm)	104.67 ± 14.19	102.84 ± 13.17	105.77 ± 15.06	105.43 ± 14.20	0.107
SBP (mmHg)	130.45 ± 20.19	129.15 ± 20.94	133.31 ± 19.96	128.95 ± 19.44	0.077
DBP (mmHg)	71.70 ± 9.39	70.91 ± 10.15	71.91 ± 8.65	72.29 ± 9.29	0.366
eGFR (mL/min/1.73 m2)	91.86 ± 20.19	88.19 ± 16.99	89.37 ± 19.02	98.06 ± 22.79	** < 0.001**
FPG (mg/dl)	**150.22 ± 53.21**	**127.13 ± 31.31**	**142.42 ± 39.19**	**181.46 ± 66.68**	** < 0.001**
FPG at phase 3 (mg/dl)	**153.43 ± 58.79**	**126.86 ± 30.96**	**141.61 ± 36.31**	**192.23 ± 76.08**	** < 0.001**
FPG at phase 4 (mg/dl)	**141.00 ± 50.74**	**127.35 ± 30.08**	**137.37 ± 39.12**	**158.49 ± 69.49**	** < 0.001**
Anti-diabetic drug at baseline	390 (74.86)	128 (72.73)	134 (77.91)	128 (73.99)	0.511
	Without diabetes (*n* = 3,769)	T1 (*n* = 1,278)	T2 (*n* = 1,253)	T3 (*n* = 1,238)	*P* value
Age (year)	**60.41 ± 9.89**	**60.78 ± 9.83**	**60.62 ± 9.91**	**59.83 ± 9.90**	**0.036**
Sex (women)	1,968 (52.22)	685 (53.60)	631 (50.36)	652 (52.67)	0.245
Marital status (married)	2,394 (63.52)	804 (62.91)	815 (65.04)	775 (62.60)	0.385
Education (high school and more)	2,630 (69.78)	885 (69.25)	873 (69.67)	872 (70.44)	0.806
Antihypertensive drug	1,059 (28.10)	349 (27.31)	338 (26.98)	372 (30.05)	0.173
Lipid-lowering drug	490 (13.00)	154 (12.05)	159 (12.69)	177 (14.30)	0.227
Current smoking	**1,858 (49.30)**	**591 (46.24)**	**642 (51.24)**	**625 (50.48)**	**0.025**
Moderate-high physical activity	2321 (61.58)	797 (62.36)	763 (60.89)	761 (61.47)	0.746
BMI (Kg/m^2^)	**27.57 ± 4.99**	**27.08 ± 4.80**	**27.40 ± 4.85**	**28.24 ± 5.25**	** < 0.001**
WC (cm)	**95.77 ± 13.52**	**94.56 ± 12.95**	**95.45 ± 13.27**	**97.34 ± 14.18**	** < 0.001**
SBP (mmHg)	122.92 ± 20.21	123.13 ± 20.37	122.65 ± 19.95	122.97 ± 20.31	0.834
DBP (mmHg)	71.55 ± 10.03	71.63 ± 10.00	71.25 ± 9.84	71.79 ± 10.24	0.388
eGFR (mL/min/1.73 m2)	82.29 ± 13.76	82.31 ± 13.56	82.14 ± 13.40	82.42 ± 14.34	0.875
FPG (mg/dl)	87.51 ± 8.90	88.80 ± 7.45	87.67 ± 8.38	87.06 ± 10.63	0.089
FPG at phase 3 (mg/dl)	**90.64 ± 8.58**	**88.56 ± 7.17**	**89.88 ± 7.63**	**93.57 ± 9.83**	** < 0.001**
FPG at phase 4 (mg/dl)	**90.22 ± 8.71**	**88.39 ± 7.33**	**89.81 ± 8.18**	**92.52 ± 9.96**	** < 0.001**

Data are represented as mean ± standard deviation for continuous variables and frequency (percent) for categorical variables

*T *tertile, *CVD *cardiovascular disease, *BMI *body mass index, *WC *waist circumference, *SBP *systolic blood pressure, *DBP* diastolic blood pressure, *eGFR* estimated glomerular filtration rate, *FPG* fasting plasma glucose

After a median follow-up of 9.2 years (interquartile range: 6.6–9.6 years), 106 incident eGFR decline ≥ 30% and 49 incident eGFR decline ≥ 40% among subjects with T2D occurred; the corresponding values for non-diabetic participants were, 157 and 62, respectively.

Table 5 shows the association of FPG variability as a continuous variable in four models with an eGFR decline ≥ 40%. Among participants with T2D, each unit change in FPG variability measures was significantly associated with a higher risk for eGFR decline in model 4 of FPG-SD, FPG-CV, and FPG-VIM; the corresponding HRs and 95% CIs were 1.01 (1.00–1.02), 1.02 (1.00–1.03), and 1.01(1.00–1.02), respectively. However, among participants without T2D, none of these measures were associated with an eGFR decline ≥ 40%. The associations of FPG variability as a categorical variable with incident eGFR decline ≥ 40% are presented in Table 6. As shown in Table 6, a significant increasing trend was observed for incident eGFR decline among participants with T2D for FPG-SD in model 1, and FPG-ARV in both models 1 and 3. Furthermore, the third tertiles of FPG-SD (model 1), and FPG-ARV (models 1 and 3) were associated with significantly higher risks, the corresponding HRs and 95% CIs were 2.15 (1.03–4.50), 2.45 (1.16–5.16), and 2.31 (1.08–4.96), respectively. However, no trends were observed in participants without T2D.

**Table 5:** HRs and 95% CIs of incident eGFR decline ≥ 40% according to each unit increase in FPG variability measures in Multi-Ethnic Study of Atherosclerosis

Variability measures	**Model 1**		**Model 2**		**Model 3**		**Model 4**	
	HR (95% CI)	*P* value	HR (95% CI)	*P* value	HR (95% CI)	*P* value	HR (95% CI)	*P *value
SD								
With diabetes	1.01(1.01–1.02)	**0.001**	1.01(1.00–1.02)	**0.040**	1.01(1.01–1.02)	**0.002**	1.01 (1.00–1.02)	**0.042**
Without diabetes	1.03(0.94–1.12)	0.516	1.02(0.94–1.11)	0.615	1.01(0.93–1.10)	0.762	1.01(0.93–1.10)	0.790
CV								
With diabetes	1.02 (1.01–1.04)	**0.008**	1.02(1.00–1.03)	0.067	1.02(1.00–1.03)	**0.020**	1.02 (1.00–1.03)	**0.041**
Without diabetes	1.02(0.94–1.10)	0.635	1.02(0.94–1.10)	0.631	1.01(0. 93–1.09)	0.797	1.01(0.93–1.09)	0.799
ARV								
With diabetes	1.01(1.00–1.01)	**0.006**	1.01(1.00–1.01)	**0.026**	1.01(1.00–1.01)	**0.007**	1.01 (1.00–1.01)	0.087
Without diabetes	1.00(0.94–1.07)	0.889	1.00(0.94–1.07)	0.990	0.99(0.93–1.06)	0.827	0.99(0.93–1.06)	0.794
VIM								
With diabetes	1.01(1.00–1.02)	0.052	1.00(1.01–1.02)	0.119	1.01(1.00–1.02)	0.144	1.01(1.00–1.02)	0.055
Without diabetes	1.02(0.93–1.11)	0.656	1.02(0.93–1.12)	0.633	1.01(0.93–1.10)	0.803	1.01(0.93–1.10)	0.799

Model 1: adjusted for age and sex at baseline (phase 2)

Model 2: Model 1 + marital status, education, ever smoking, prevalent CVD, physical activity, anti-diabetic drug use, anti-hypertensive drug, lipid-lowering drug, BMI, WC, SBP, DBP, eGFR, and FPG at baseline (phase 2)

Model 3: Model 1 + marital status, education, ever smoking, prevalent CVD, and physical activity at baseline (phase 2), and anti-diabetic drug use, anti-hypertensive drug, and lipid-lowering drug over phases 2–4, and average BMI, WC, SBP, DBP, and eGFR over phases 2–4

Model 4: Model 3 + average FPG

**Table 6:** HRs and 95% CIs of incident eGFR decline ≥ 40% according to tertiles of FPG variability measures in Multi-Ethnic Study of Atherosclerosis

Variability measures	**Model 1**	**Model 2**	**Model 3**	**Model 4**
	HR (95% CI)	HR (95% CI)	HR (95% CI)	HR (95% CI)
**With diabetes**				
SD				
T1	1.00 (reference)	1.00 (reference)	1.00 (reference)	1.00 (reference)
T2	1.48 (0.69–3.19)	1.28 (0.58–2.81)	1.26 (0.57–2.77)	1.18 (0.53–2.60)
T3	**2.15 (1.03–4.50)**	1.38 (0.59–2.22)	1.96 (0.93–4.17)	1.46 (0.64–3.34)
P _trend_	**0.040**	0.459	0.069	0.367
CV				
T1	1.00 (reference)	1.00 (reference)	1.00 (reference)	1.00 (reference)
T2	0.81 (0.37–1.79)	0.65 (0.29–1.49)	0.70 (0.31–1.57)	0.66 (0.29–1.49)
T3	1.78 (0.91–3.47)	1.32 (0.65–2.70)	1.60 (0.81–3.16)	1.35 (0.67–2.75)
P _trend_	0.073	0.326	0.128	0.307
ARV				
T1	1.00 (reference)	1.00 (reference)	1.00 (reference)	1.00 (reference)
T2	1.48(0.67–3.30)	1.16 (0.50–2.68)	1.35 (0.60–3.06)	1.20 (0.53–2.75)
T3	**2.45 (1.16–5.16)**	1.79 (0.79–4.08)	**2.31 (1.08–4.96)**	1.78 (0.78–4.06)
P _trend_	**0.015**	0.132	**0.025**	0.145
VIM				
T1	1.00 (reference)	1.00 (reference)	1.00 (reference)	1.00 (reference)
T2	0.54 (0.24–1.23)	0.51 (0.22–1.18)	0.50 (0.21–1.13)	0.48 (0.21–1.12)
T3	1.52 (0.80–2.87)	1.39 (0.72–2.69)	1.35 (0.70–2.61)	1.49 (0.76–2.93)
P _trend_	0.155	0.234	0.264	0.178
**Without diabetes**				
SD				
T1	1.00 (reference)	1.00 (reference)	1.00 (reference)	1.00 (reference)
T2	0.57 (0.30–1.09)	0.59 (0.31–1.14)	0.59(0.31–1.13)	0.59 (0.30–1.13)
T3	0.93 (0.53–1.64)	0.95 (0.53–1.68)	0.88 (0.49–1.56)	0.87 (0.49–1.55)
P _trend_	0.744	0.804	0.623	0.603
CV				
T1	1.00 (reference)	1.00 (reference)	1.00 (reference)	1.00 (reference)
T2	0.74 (0.40–1.36)	0.79 (0.43–1.45)	0.76 (0.41–1.40)	0.76 (0.41–1.40)
T3	0.82 (0.45–1.49)	0.86 (0.47–1.58)	0.80 (0.44–1.46)	0.80 (0.44–1.46)
P _trend_	0.483	0.600	0.445	0.444
ARV				
T1	1.00 (reference)	1.00 (reference)	1.00 (reference)	1.00 (reference)
T2	0.77 (0.42–1.39)	0.74 (0.41–1.35)	0.74 (0.41–1.35)	0.74 (0.41–1.34)
T3	0.70 (0.38–1.30)	0.69 (0.37–1.28)	0.65 (0.35–1.21)	0.64 (0.34–1.19)
P _trend_	0.243	0.215	0.160	0.148
VIM				
T1	1.00 (reference)	1.00 (reference)	1.00 (reference)	1.00 (reference)
T2	0.67 (0.36–1.24)	0.73 (0.39–1.36)	0.70 (0.38–1.30)	0.70 (0.38–1.29)
T3	0.78 (0.43–1.41)	0.83 (0.45–1.52)	0.77 (0.43–1.40)	0.77 (0.43–1.41)
P _trend_	0.384	0.516	0.372	0.375

Model 1: adjusted for age and sex at baseline (phase 2)

Model 2: Model 1 + marital status, education, ever smoking, prevalent CVD, physical activity, anti-diabetic drug use, anti-hypertensive drug, lipid-lowering drug, BMI, WC, SBP, DBP, eGFR, and FPG at baseline (phase 2)

Model 3: Model 1 + marital status, education, ever smoking, prevalent CVD, and physical activity at baseline (phase 2), and anti-diabetic drug use, anti-hypertensive drug, and lipid-lowering drug over phases 2–4, and average BMI, WC, SBP, DBP, and eGFR over phases 2–4

Model 4: Model 3 + average FPG

The association between FPG variability measures and eGFR decline ≥ 30% was in line with the reported results for GFR decline ≥ 40%, except that one unit increase in FPG-ARV was significantly associated with a higher risk for eGFR decline in model 4 among T2D participants (1.01 (1.00–1.01)) (Supplementary Tables 8 and 9).

### Pooled data of TLGS and MESA cohorts

The association between FPG variability measures and eGFR decline ≥ 40% as a continuous and categorical variable was also measured in pooled data of MESA and TLGS cohorts (Tables 7 and 8, respectively). Among participants without T2D, each unit change in FPG variability measures was significantly associated with a higher risk for eGFR decline in all models of FPG-SD, FPG-CV, and FPG-VIM; the corresponding HRs and 95% CIs of model 4 were 1.05 (1.01–1.10), 1.05 (1.00–1.09), and 1.05 (1.00–1.10), respectively. Among participants with T2D, only model 1 of FPG-ARV was associated with eGFR decline ≥ 40% (1.01(1.00–1.01)). However, no association was shown between FPG variability as a categorical variable and risk of eGFR decline in any of the models in both individuals with and without T2D in pooled data of TLGS and MESA cohorts. When we replaced eGFR decline ≥ 30% in place of eGFR ≥ 40% as the outcome of the study, no significant association was demonstrated for FPG variability measures in the fully adjusted model in both individuals and without T2D in the pooled data of two cohorts (data not shown).

**Table 7:** HRs and 95% CIs of incident eGFR decline ≥ 40% according to each unit increase in FPG variability measures in pooled data of Tehran Lipid and Glucose Study and Multi-Ethnic Study of Atherosclerosis cohorts

Variability measures	**Model 1**		**Model 2**		**Model 3**		**Model 4**	
	HR (95% CI)	*P* value	HR (95% CI)	*P* value	HR (95% CI)	*P* value	HR (95% CI)	*P* value
SD								
With diabetes	1.01(1.00–1.01)	0.054	1.00 (0.99–1.01)	0.767	1.00 (1.00–1.01)	0.175	1.00 (0.99–1.01)	0.881
Without diabetes	**1.06(1.01–1.11)**	**0.010**	**1.05(1.01–1.10)**	**0.025**	**1.05 (1.01–1.10)**	**0.024**	**1.05 (1.01–1.10)**	**0.025**
CV								
With diabetes	1.01 (1.00–1.02)	0.147	1.00 (0.99–1.01)	0.799	1.00 (0.99–1.02)	0.394	1.00 (0.99–1.02)	0.725
Without diabetes	**1.05(1.01–1.09)**	**0.020**	**1.04 (1.00–1.09)**	**0.038**	**1.05 (1.00–1.09)**	**0.030**	**1.05 (1.00–1.09)**	**0.030**
ARV								
With diabetes	**1.01(1.00–1.01)**	**0.026**	1.00 (1.00–1.01)	0.411	1.00 (1.00–1.01)	0.105	1.00 (1.00–1.01)	0.590
Without diabetes	1.02(0.99–1.06)	0.248	1.02 (1.09–1.06)	0.231	1.02 (0.98–1.05)	0.323	1.02 (0.98–1.05)	0.343
VIM								
With diabetes	1.00 (1.00–1.01)	0.438	1.00 (0.99–1.01)	0.806	1.00 (0.99–1.01)	0.825	1.00 (0.99–1.01)	0.580
Without diabetes	**1.05(1.01–1.10)**	**0.025**	**1.05 (1.00–1.10)**	**0.043**	**1.05 (1.00–1.10)**	**0.032**	**1.05 (1.00–1.10)**	**0.032**

Model 1: adjusted for age and sex at baseline (phase 2)

Model 2: Model 1 + marital status, education, ever smoking, prevalent CVD, physical activity, anti-diabetic drug use, anti-hypertensive drug, lipid-lowering drug, BMI, WC, SBP, DBP, eGFR, and FPG at baseline (phase 2)

Model 3: Model 1 + marital status, education, ever smoking, prevalent CVD, and physical activity at baseline (phase 2), and anti-diabetic drug use, anti-hypertensive drug, and lipid-lowering drug over phases 2–4, and average BMI, WC, SBP, DBP, and eGFR over phases 2–4

Model 4: Model 3 + average FPG

**Table 8:** HRs and 95% CIs of incident eGFR decline ≥ 40% according to tertiles of FPG variability measures in pooled data of Tehran Lipid and Glucose Study and Multi-Ethnic Study of Atherosclerosis cohorts

Variability measures	**Model 1**	**Model 2**	**Model 3**	**Model 4**
	HR (95% CI)	HR (95% CI)	HR (95% CI)	HR (95% CI)
**With diabetes**				
SD				
T1	1.00 (reference)	1.00 (reference)	1.00 (reference)	1.00 (reference)
T2	1.29 (0.82–2.02)	1.05 (0.66–1.66)	1.08 (0.69–1.71)	1.00 (0.63–1.59)
T3	1.41 (0.89–2.21)	0.92 (0.56–1.52)	1.19 (0.75–1.90)	0.92 (0.55–1.56)
P _trend_	0.141	0.734	0.462	0.766
CV				
T1	1.00 (reference)	1.00 (reference)	1.00 (reference)	1.00 (reference)
T2	0.95 (0.60–1.49)	0.75 (0.47–1.19)	0.80 (0.50–1.26)	0.74 (0.47–1.18)
T3	1.27 (0.83–1.95)	0.92 (0.59–1.44)	1.07 (0.69–1.65)	0.93 (0.59–1.47)
P _trend_	0.268	0.788	0.730	0.837
ARV				
T1	1.00 (reference)	1.00 (reference)	1.00 (reference)	1.00 (reference)
T2	1.45 (0.92–2.29)	1.09 (0.68–1.76)	1.13 (0.71–1.81)	1.05 (0.65–1.69)
T3	1.58 (0.99–2.49)	1.08 (0.65–1.79)	1.31 (0.82–2.11)	1.04 (0.62–1.76)
P _trend_	0.055	0.781	0.253	0.885
VIM				
T1	1.00 (reference)	1.00 (reference)	1.00 (reference)	1.00 (reference)
T2	0.87 (0.55–1.37)	0.84 (0.53–1.33)	0.83 (0.52–1.31)	0.84 (0.53–1.33)
T3	1.19 (0.78–1.81)	1.06 (0.69–1.62)	1.05 (0.68–1.62)	1.11 (0.72–1.71)
P _trend_	0.423	0.779	0.805	0.629
**Without diabetes**				
SD				
T1	1.00 (reference)	1.00 (reference)	1.00 (reference)	1.00 (reference)
T2	0.94 (0.65–1.37)	0.99 (0.68–1.45)	0.96 (0.66–1.41)	0.96 (0.66–1.41)
T3	1.28 (0.90–1.82)	1.32 (0.93–1.88)	1.26 (0.88–1.79)	1.25 (0.88–1.79)
P _trend_	0.161	0.123	0.197	0.208
CV				
T1	1.00 (reference)	1.00 (reference)	1.00 (reference)	1.00 (reference)
T2	1.07 (0.74–1.54)	1.13 (0.78–1.63)	1.12 (0.77–1.62)	1.12 (0.77–1.62)
T3	1.20 (0.84–1.73)	1.25 (0.87–1.79)	1.20 (0.84–1.73)	1.20 (0.84–1.72)
P _trend_	0.310	0.235	0.320	0.324
ARV				
T1	1.00 (reference)	1.00 (reference)	1.00 (reference)	1.00 (reference)
T2	0.94 (0.65–1.34)	0.99 (0.69;1.42)	0.99 (0.69–1.42)	0.98 (0.68–1.42)
T3	1.02 (0.72–1.45)	1.06 (0.75–1.51)	1.02 (0.71–1.45)	1.01 (0.71–1.44)
P _trend_	0.918	0.743	0.938	0.973
VIM				
T1	1.00 (reference)	1.00 (reference)	1.00 (reference)	1.00 (reference)
T2	1.06 (0.73–1.53)	1.12 (0.78–1.63)	1.11 (0.77–1.61)	1.11 (0.77–1.61)
T3	1.24 (0.87–1.78)	1.28 (0.89–1.84)	1.25 (0.87–1.79)	1.25 (0.87–1.80)
P _trend_	0.234	0.184	0.224	0.223

Model 1: adjusted for age and sex at baseline (phase 2)

Model 2: Model 1 + marital status, education, ever smoking, prevalent CVD, physical activity, anti-diabetic drug use, anti-hypertensive drug, lipid-lowering drug, BMI, WC, SBP, DBP, eGFR, and FPG at baseline (phase 2)

Model 3: Model 1 + marital status, education, ever smoking, prevalent CVD, and physical activity at baseline (phase 2), and anti-diabetic drug use, anti-hypertensive drug, and lipid-lowering drug over phases 2–4, and average BMI, WC, SBP, DBP, and eGFR over phases 2–4

Model 4: Model 3 + average FPG

## Discussion

For the first time, we examined the association between GV over 6 years assessed by SD, CV, ARV, and VIM and incident eGFR decline in both T2D and non-T2D individuals, separately in two well-known cohorts, namely TLGS and MESA during one a decade follow-up. Using eGFR decline ≥ 40% as the outcome, in the MESA, in the fully adjusted model, higher GV using SD and CV measures were significantly associated with a higher risk of eGFR decline among those with T2D; however, among those without T2D, no associations were found. In the TLGS, the higher GV using SD, CV, and VIM measures were significantly associated with a higher risk of eGFR decline only among those without T2D. Applying eGFR decline ≥ 30% as the outcome, in the TLGS, regardless of diabetes status, no association was shown between FPG variability measures and risk of eGFR decline; however, in the MESA the results were in line with those of GFR decline ≥ 40%. Moreover, using pooled data from the two cohorts we found that each unit increase in FPG variability with all GV measurements excluding ARV were associated with about 5% higher risk of eGFR decline ≥ 40% only among non-T2D individuals.

To the best of our knowledge, several studies [42] have assessed the relationship between GV and incident CKD, ESRD, or diabetic kidney disease (DKD) and eGFR rate decline among T2D patients. GV was assessed by HbA1c variability in all of these studies except two studies [43, 44] that were assessed by both HbA1c and FPG variability.

Currently, eGFR decline is considered a validated surrogate endpoint for ESRD in randomized clinical trials (RCTs) as well as in cohort studies [24, 45, 46]. It was shown that while 20% and 30% eGFR decline are extremely susceptible to the existence of acute effects, 40% and 57% are more robust [25]. In an international meta-analysis of more than 1.7 million individuals with incident 12,344 ESRD events, it was shown that a decline in eGFR ≥ 30% and ≥ 40% over a 3-year baseline period was associated with an adjusted HR of 7.0 (3.9–12.7) and 15.7 (7.4–33.4), respectively, compared to no changes in eGFR in those with baseline eGFR ≥ 60 mL/min/1.73 m^2^ [24]. The corresponding values for T2D Japanese patients were estimated at 18.4 (7.6–44.7) and 12.8 (5.2–32.2) [45].

The following studies [30–32], assessed GV and eGFR decline among T2D patients. All of these studies evaluated the annual changes in eGFR among T2D individuals. Takenouchi et al. found that higher HbA1C-CV was associated with a higher risk of eGFR decline, mainly among those with an albumin/creatinine ratio ≥ 30 mg/g. In this study, no association was found between FPG -GV and eGFR decline [32]. Based on eGFR decline, Low et al. also found that renal disease progression was more common among those with T2D with higher HbA1C-CV independent of mean HbA1C. However, compared with patients with better average glycemic control, T2D patients with sub-optimal average glycemic control (i.e. HbA1C > 8.0%)were more likely to develop renal disease at lower magnitudes of HbA1C variability [31]. However, Lin Lee et al. found that even among T2D patients with well-controlled HbA1C levels (< 7%), those with high HbA1C-CV still experienced faster eGFR decline[30]. In our data analysis, among T2D patients in MESA, with sub-optima mean FPG level about of 151 mg/dl, FPG variability had a greater likelihood for eGFR decline ≥ 30 and ≥ 40%.

To the best of our knowledge, there is also only one study that investigates the correlation between VVV and macrovascular and microvascular events in the general population [14]. In a 10-year prospective cohort study, Jang et al. found that the HbA1C-CV tertile was associated with an increased risk for macro-and microvascular events in non-DM middle-aged participants. The higher HbA1C variability was an independent risk factor for microvascular events, however for macrovascular events, the risk was more prominent for variabilities in FPG and post 2-h blood glucose [14]. Our findings in MESA among non-T2D participants are consistent with this study, which found no associations between FPG variability measurements and eGFR decline ≥ 40%; however, FPG-SD, FPG-CV, and FPG-VIM were associated with higher eGFR decline ≥ 40% among non-T2D participants in TLGS.

Several studies have investigated the impact of ethnicity on eGFR decline [47], progression to ESRD, and development of CKD [48]. Peralta et al. assessed the ethnicity and racial disparities in kidney function decline in participants without CKD [47]. In age- and sex-adjusted models, black individuals had a higher risk of incident CKD among those with eGFR higher than 90 ml/min per 1.73 m^2^ as well as 60 < eGFR ≤ 90 ml/min per 1.73 m^2^, followed by Hispanics, while Chinese with 60 < eGFR ≤ 90 ml/min per 1.73 m2 had the lowest risk of incident CKD. The associations attenuated following adjustment for CKD risk factors, particularly hypertension and diabetes. Therefore, the authors concluded that the rate of kidney function decline before incident CKD could be different among various ethnicities, which cannot be fully explained by differences in CKD well-established risk factors [47]. The ethnicity disparity in CKD development in diabetic patients has also been revealed. Hull et al. found a higher rate of CKD development in South Asia relative to the White population [49]. Collectively, ethnicity can influence the rate of kidney function decline and its associations with different risk factors, which could be a reasonable explanation for our different association between GV and eGFR decline in the American versus Iranian cohort. Despite our best efforts, no previous studies addressed the impact of GV variability on eGFR decline in both T2D and non-T2D simultaneously.

From a pathophysiological perspective, although several potential mechanisms have been proposed to have the potential to connect enhanced GV with a higher risk of incident micro- and macro-vascular complications of diabetes including renal impairment, ESRD, and CKD, the exact mechanism has yet to be determined. There is a bulk of evidence [9, 50–52] in support of the fact that short-term, as well as long-term GV can enhance inflammatory cytokines [53], oxidative stress[53], and induce endothelial dysfunction[44, 54–56], all of which have been shown to have mandatory roles in diabetes complications. GV can increase human tubule-interstitial cell growth, collagen production, and endothelial apoptosis rates compared with persistent exposure to high glucose levels [55, 57, 58].

### Strengths and limitations

The present study contains strengths that worth to be acknowledging. The primary strength of our study is that we examined the impact of exposure to GV on incident eGFR decline in two well-known cohorts among both participants with and without T2D during a long-term follow-up. Second, although the level of adjustment for potential risk factors is different among studies, we adjusted for well-known CKD risk factors in the current study.

On the other hand, several limitations should be mentioned. First, we did not have access to the data on HbA1C variability. Of note, Yang et al. demonstrated that both FPG-CV and HbA1c-CV can predict the development of ESRD in diabetes [44]. Furthermore, Jang et al. found out that while HbA1C variability is a better predictor of insulin resistance and inflammatory responses, FPG and 2-HPG are better predictors of sympathoadrenal activation, which was shown to be associated with hypoglycemia [14]. Second, we also did not have access to the data on the urine sample and the albuminuria status. Moreover, it was shown that eGFR is unreliable in detecting renal function in those with diabetes as it overestimates and underestimates measured GFR (mGFR) at lower mGFR and higher mGFR values, respectively [59]; thus, we used eGFR decline as a valid surrogate for renal failure similar to many RCTs [46] and few cohort studies [24, 46, 60]. Third, our study lacked data concerning the episodes of hypoglycemia, which was revealed to enhance the risk of CKD in those with T2D [61]. Forth, we removed participants with eGFR decline ≥ 30% during the period of FPG fluctuations; however, since our exposure period did not proceed outcome in the strict sense (i.e. the time of start for eGFR decline and FPG variability assessment was the same), the absence of mentioned time lag may lead to inverse causality. Finally, the TLGS cohort was performed among residents of the metropolitan city of Tehran; thus, our findings cannot be extrapolated into rural zones of Iran and other ethnicities. It is important to note that the MESA cohort represents four different ethnicities including the white population which constitute the greatest part of the cohort (about 40%) followed by African American, Hispanic and Chinese Americans, aged ≥ 45 years [62], so it cannot be extrapolated to the younger age population.

## Conclusion

In summary, we found that higher FPG variability is associated with an increased risk of eGFR decline of ≥ 30 and ≥ 40% in the American population with diabetes. However, the unfavorable impact of FPG-GV was found only among the non-diabetic Iranian population for incident eGFR decline of ≥ 30%.

## Supplementary Information

**Additional file 1:Supplementary Table 1.** HRs and 95% CIs of incident eGFR decline ≥ 30% according to each unit increase in FPG variability measures in Tehran Lipid and Glucose Study. **Supplementary Table 2.** HRs and 95% CIs of incident eGFR decline ≥ 30% according to tertiles of FPG variability measures in Tehran Lipid and Glucose Study. **Supplementary Table 3.** HRs and 95% CIs of incident eGFR decline ≥ 30% according to each unit increase in 2-HPG variability measures in Tehran Lipid and Glucose Study. **Supplementary Table 4.** HRs and 95% CIs of incident eGFR decline ≥ 30% according to tertiles of 2-HPG variability measures in Tehran Lipid and Glucose Study. **Supplementary Table 5.** HRs and 95% CIs of incident eGFR decline ≥ 40% according to each unit increase 2-HPG variability measures in Tehran Lipid and Glucose Study. **Supplementary Table 6.** HRs and 95% CIs of incident eGFR decline ≥ 40% according to each unit increase 2-HPG variability measures in Tehran Lipid and Glucose Study. **Supplementary Table 7.** HRs and 95% CIs of incident eGFR decline ≥ 30% and 40% according to each unit increase in 2-HPG variability measures in Tehran Lipid and Glucose Study. **Supplementary Table 8.** HRs and 95% CIs of incident eGFR decline ≥ 30% according to each unit increase in FPG variability measures in Multi-Ethnic Study of Atherosclerosis. **Supplementary Table 9.** HRs and 95% CIs of incident eGFR decline ≥ 30% according to tertiles of FPG variability measures in Multi-Ethnic Study of Atherosclerosis.

## Data Availability

The data sets used and/or analyzed during the current study are available from the corresponding author upon reasonable request.
