# Supplementary material for: The association between fasting plasma glucose variability and incident eGFR decline: evidence from two cohort studies
Source: BMC Public Health. 2023 Mar 27;23:565. doi: 10.1186/s12889-023-15463-8 (PMC10041700; doi:10.1186/s12889-023-15463-8)
Supplement: Supplementary file 1 — Additional file 1:Supplementary Table 1. HRs and 95% CIs of incident eGFR decline ≥ 30% according to each unit increase in FPG variability measures in Tehran Lipid and Glucose Study. Supplementary Table 2. HRs and 95% CIs of incident eGFR decline ≥ 30% according to tertiles of FPG variability measures in Tehran Lipid and Glucose Study. Supplementary Table 3. HRs and 95% CIs of incident eGFR decline ≥ 30% according to each unit increase in 2-HPG variability measures in Tehran Lipid and Glucose Study. Supplementary Table 4. HRs and 95% CIs of incident eGFR decline ≥ 30% according to tertiles of 2-HPG variability measures in Tehran Lipid and Glucose Study. Supplementary Table 5. HRs and 95% CIs of incident eGFR decline ≥ 40% according to each unit increase 2-HPG variability measures in Tehran Lipid and Glucose Study. Supplementary Table 6. HRs and 95% CIs of incident eGFR decline ≥ 40% according to each unit increase 2-HPG variability measures in Tehran Lipid and Glucose Study. Supplementary Table 7. HRs and 95% CIs of incident eGFR decline ≥ 30% and 40% according to each unit increase in 2-HPG variability measures in Tehran Lipid and Glucose Study. Supplementary Table 8. HRs and 95% CIs of incident eGFR decline ≥ 30% according to each unit increase in FPG variability measures in Multi-Ethnic Study of Atherosclerosis. Supplementary Table 9. HRs and 95% CIs of incident eGFR decline ≥ 30% according to tertiles of FPG variability measures in Multi-Ethnic Study of Atherosclerosis. [file 12889_2023_15463_MOESM1_ESM.docx]

**The association between fasting plasma glucose variability and incident eGFR decline: evidence from two cohort studies**

**Running title: Glucose variability and eGFR decline**

Niloofar Deravi^1,2+^, Yasaman Sharifi^1,3+^, Fatemeh Koohi^1^, Seyed Saeed Tamehri Zadeh^1^, Soroush Masrouri^1^, Fereidoun Azizi^4^, and Farzad Hadaegh^1*^

1. Prevention of Metabolic Disorders Research Center, Research Institute for Endocrine Sciences, Shahid Beheshti University of Medical Sciences, Tehran, Iran.
2. Student Research Committee, School of Medicine, Shahid Beheshti University of Medical Sciences, Tehran, Iran.
3. Department of Radiology, School of Medicine, Iran University of Medical Sciences, Tehran, Iran.
4. Endocrine Research Center, Research Institute for Endocrine Sciences, Shahid Beheshti University of Medical Sciences, Tehran, Iran.

^*^ Corresponding author:

Farzad Hadaegh, MD. Prevention of Metabolic Disorders Research Center, Research Institute for Endocrine Sciences, Shahid Beheshti University of Medical Sciences. No. 24, Parvaneh Street, Velenjak, Tehran, Iran.

P.O. Box: 19395-4763

Phone: +98 21 22432500

Email: [fzhadaegh@endocrine.ac.ir](mailto:fzhadaegh@endocrine.ac.ir)

**+: Yasaman Sharifi and Niloofar Deravi contributed equally to this work and were co-first authors.**

**Manuscript word count: 4554**

**Number of tables: 8**

**Number of figures: 2**

**Number of supplementary digital content files: 9 Tables**

**Abstract**

**Background**

Glycemic variability (GV) is developing as a marker of glycemic control, which can be utilized as a promising predictor of complications. To determine whether long-term GV is associated with incident eGFR decline in two cohorts of Tehran Lipid and Glucose Study (TLGS) and Multi-Ethnic Study of Atherosclerosis (MESA) during a median follow-up of 12.2 years.

**Methods**

Study participants included 4422 Iranian adults (including 528 patients with T2D) aged ≥ 20 years from TLGS and 4290 American adults (including 521 patients with T2D) aged ≥45 years from MESA. The Multivariate Cox proportional hazard models were used to assess the risk of incident eGFR decline for each of the fasting plasma glucose (FPG) variability measures including standard deviation (SD), coefficient of variation (CV), average real variability (ARV), and variability independent of the mean (VIM) both as continuous and categorical variables. The time of start for eGFR decline and FPG variability assessment was the same, but the event cases were excluded during the exposure period.

**Results**

In TLGS participants without T2D, for each unit change in FPG variability measures, the hazards (HRs) and 95% confidence intervals (CI) for eGFR decline ≥40% of SD, CV, and VIM were 1.07(1.01-1.13), 1.06(1.01-1.11), and 1.07(1.01-1.13), respectively. Moreover, the third tertile of FPG-SD and FPG-VIM parameters was significantly associated with a 60 and 69% higher risk for eGFR decline ≥40%, respectively. In MESA participants with T2D, each unit change in FPG variability measures was significantly associated with a higher risk for eGFR decline ≥40%.

Regarding eGFR decline **≥**30% as the outcome, in the TLGS, regardless of diabetes status, no association was shown between FPG variability measures and risk of eGFR decline in any of the models; however, in the MESA the results were in line with those of GFR decline **≥**40%.

Using pooled data from the two cohorts we found that generally FPG variability were associated with higher risk of eGFR decline **≥**40% only among non-T2D individuals.

**Conclusions**

Higher FPG variability was associated with an increased risk of eGFR decline in the diabetic American population; however, this unfavorable impact was found only among the non-diabetic Iranian population.

**Keywords:** **Glycemic variability,** **Fasting plasma glucose,** **Type 2 diabetes,** **Estimated glomerular filtration rate decline, eGFR decline.**

**Introduction**

Glycemic variability (GV), also known as blood glucose swings, includes a wide range of blood glucose variations that occur throughout the day, including hypoglycemic periods and postprandial spikes, as well as fluctuations that occur at the same time on different days. Diabetes complications are associated with GV in both type one diabetes and type 2 diabetes (T2D) patients [1-5]. Accordingly, GV was shown to be mostly associated with oxidative stress [6] and an increased incidence of hypoglycemia, a trigger of inflammatory processes [5, 7]. GV is measured on a short- and long-term basis [5]. Evidence supports the importance of this index both as a criterion for glycemic control and in the prevention of complications among patients with diabetes [1, 2, 4, 5, 8-12].

Short-term GV refers to between-days or within-day glycemic fluctuations, which are often measured via continuous glucose monitoring mostly in patients with type 1 diabetes [13]. Long-term GV refers to glycemic fluctuations over months to years, mostly measured by visit-to-visit variability in either fasting plasma glucose (FPG) or HbA1c in patients with diabetes [13]. Previous studies have shown that long-term GV may predict chronic kidney disease (CKD), the main microvascular complication of diabetes leading to both morbidity and mortality [14-18] with a high prevalence and incidence rate among both Iranian and American populations [19-22].The Food and Drug Administration (FDA) committee and National Kidney Foundation (NKF) have published a series of studies to investigate whether eGFR declines less than 50% could be defined as an important kidney endpoint [24-27]. The committee reported that a 30% decline in eGFR can be regarded as a reliable surrogate endpoint in some circumstances; however, a 40% eGFR decline could present stronger evidence [28-30]. Accordingly, a meta-analysis of 1.7 million participants reported the average adjusted 10-year risk of end-stage renal disease (ESRD) for eGFR declines of 40% and 30%, were 83% and 64%, respectively [25].

 Previous studies mainly conducted among T2D patients in the East Asian region have investigated the effect of HbA1c variation on eGFR decline [31-33], however, there is a paucity of information on the effect of FPG variability on eGFR decline in individuals with T2D [33]. Therefore, the current study for the first time investigated the association of long-term FPG variability with eGFR decline **≥** 30 and 40% in non-CKD adults with and without T2D in the Tehran Lipid and Glucose Study (TLGS), as well as in the participants of the Multi-Ethnic Study of Atherosclerosis (MESA) study during about one decade of follow-up.

**Methods**

**2.1. Study population**

Study participants were selected from participants of TLGS, an ongoing large-scale population-based cohort study conducted on a representative population of Tehran city, the capital of Iran. TLGS aims to determine the risk factors for non-communicable diseases. The design of TLGS has been published before [34, 35]. In brief, in phase 1 (1999–2001) 15,005 participants ≥3 years entered the study; the data collection has been continued since then at approximately three-year intervals in follow-up phases (i.e., phases 2 (2002–2005), 3 (2005–2008), 4 (2008–2011), 5 (2011–2014), and 6 (2014–2017)). Moreover, 3555 participants entered the cohort in phase 2 of the study and were subsequently followed in phases 3, 4, 5, and 6.

In the present study, we included 9137 participants of the TLGS cohort (1057 patients with T2D) aged ≥ 20 years who participated in phase 2 (as the baseline phase). We aimed to calculate the visit-to-visit variability (VVV) of FPG; therefore, we included those with available FPG values in phases 3, and 4. . Consequently, we considered 2002–2011 as the exposure period. We excluded individuals with no measurement of FPG at any of the phases 2-4, diagnosed CKD before phase 2 and eGFR decline **≥** 40% during the measurement period (i.e., at any of the phases 3-4), lost to follow-up at any of the phases, and missing data on covariates. Among non-diabetes group, those with incident T2D at any of the phases 3-6 were also excluded. Finally, 4422 participants (including 528 patients with T2D) remained for data analysis and followed till March 2018. Using a similar approach, for the eGFR decline **≥** 30%, 4,181 individuals (483 patients with T2D) were entered in the data analysis. **Figure**. 1 demonstrates the flowchart of the TLGS study participants.

The MESA dataset, from a longitudinal study, was also used. Participants aged 45 to 84 years at baseline, from six sites around the United States, were oversampled by four ethnic/racial groups. Design and objectives have been described in detail elsewhere [36]. Briefly, after the baseline examination, there have been five additional follow-up visits at biennial intervals, the most recent ongoing in 2016-18. Institutional Review Board approval was granted at each site and informed consent was obtained from each participant. MESA included 6814 participants (859 patients with T2D aged ≥ 45 years who participated in phase 1 (Sep 2002 to Feb 2004). Similar to the process for the TLGS cohort we measured the VVV of FPG in MESA participants; therefore, we included those with available FPG values in sequential phases 1, 2 (Mar 2004 to Sep 2005), and 3 (2005 to May 2007). We excluded individuals with no measurement of FPG at any of the phases 1-3, diagnosed CKD at phases 1, eGFR decline **≥** 40% at any of the phases 2-3, lost follow-up at any of the phases, and missing data on covariates. Those with incident T2D at any of the phases 2-5 in the non-diabetic group were also excluded. Finally, 4290 participants (including 521 patients with T2D) remained for data analysis. Hence, the final samples were followed for incident eGFR decline **≥** 40% after the measurement period. Using the similar approach, for the eGFR decline ≥ 30%, 4,290 individuals (521 patients with T2D) were entered in the data analysis. **Figure. 2** demonstrates the flowchart of the MESA study participants.

**2.2.** **Clinical and laboratory measurements**

We collected the baseline characteristics including data on age, sex, drug, family, past medical history, and smoking status (never or ever) through a standard questionnaire and measured body weight and height, diastolic blood (DBP), and systolic (SBP) pressure during a clinical examination. We calculated body mass index (BMI) as weight (kg) divided by the square of height (m^2^). Using a standard mercury sphygmomanometer, we measured blood pressure twice in a seating position after 15 minutes of rest. We also drew blood samples for the measurement of FPG, total cholesterol (TC), and creatinine levels, after 12–14 h of overnight fasting, from all participants, assayed serum creatinine levels by the Jaffe kinetic calorimetric method, and analyzed the samples once internal quality control met the standard satisfactory criteria. The standard 2-hour post-load plasma glucose (2-HPG) test was also performed for those not on glucose lowering drugs. Physical activity level was assessed using the Modifiable Activity Questionnaire (Low, moderate-high) [37]. Moderate-high Physical activity level was defined as achieving a score ≤600 MET-minutes per week [38]. Details of laboratory assessments were reported previously [23]. Detailed description of MESA clinical and laboratory measurements has been published elsewhere [36].

We defined VVV as an intra individual variability in FPG levels recorded across three consecutive examinations in both TLGS and MESA. We used four indices of variability: (a) coefficient of variation (CV), (b) Standard deviation (SD), (c) average real variability (ARV), and (d) variability independent of the mean (VIM). We calculated VIM as 100 * SD/mean^β^, where β is the regression coefficient based on the natural logarithm of SD on the natural logarithm of the mean. We calculated ARV according to the following formula, where n denotes the number of measures of FPG [39].

ARV= $\frac{1}{n-1}\sum_{i=1}^{n-1} \left| Value(i+1 \right)-Value(i)|$

ARV is the average of absolute differences between consecutive values.

**2.3. Definition of outcomes and variables**

The main outcome for this study was a reduction in eGFR of **≥**40 and **≥**30% from the baseline. eGFR was estimated by use of the Chronic Kidney Disease Epidemiology Collaboration (CKD-EPI) equation.

141*min (Scr/ƙ, 1)α *max (Scr/ƙ , 1)-1.209 *0.993age *1.018 (if female)*1.159 (if black).

In this equation, we measured serum creatinine (Scr) in mg/dL, and age in years. ƙ is 0.9 and 0.7 for women and men, respectively; α is -0.329 and -0.411 for women and men; min indicates the minimum of Scr/ƙ or 1, and max indicates a maximum of Scr/ƙ or 1. eGFR was expressed as mL/min/1.73 m2.

The percentage of change in eGFR was calculated as:

$$\frac{Follow up measurement- Baseline measurement}{Baseline measurement} \times100$$

We defined T2D as FPG of ≥ 126 mg/dl or current use of antidiabetic drugs. Moreover, prevalent cardiovascular disease (CVD) was a self-reported CVD history with a prior diagnosis of CVD by a physician. Smoking was categorized as ever smoker (current or past) versus non-smoker. Smoking was categorized as ever smoker (current or past) versus non-smoker. Hypertension, was defined as the presence of at least one of the following criteria: (a) having SBP≥140 mm Hg, (b) having DBP≥90 mm Hg, and (c) initiation of anti-hypertensive drugs usage.

**2.4. Statistical methods**

We showed the baseline characteristics of participants as frequency (%) for categorical variables and the mean ± standard deviation (SD) for continuous variables. We also categorized participants with and without T2D into 3 groups according to the tertiles of FPG-SD and compared the baseline characteristics across these tertiles. Chi-square test and analysis of variance were used to compare the clinical and demographic characteristics, as appropriate.

The follow-up time used for time-to-event analyses was defined as the time from baseline date (phase 2) to either eGFR decline≥40 or 30% in phases 5 or 6, or date of last data collection or death, whichever occurred first. Only the first occurrence of each outcome was used for analysis.

Each glucose variability measure was categorized by tertiles with a reference level of the lowest tertile. For individuals with incident eGFR decline, survival time was defined as the mid-time between the entered date and the event date. Linear trends across the tertiles were calculated by including the tertile as a continuous variable in the models. Moreover, to estimate adjusted HRs and 95% CIs for incident eGFR decline associated with GV, we applied multivariable Cox proportional hazard (Cox PH) models, separately for participants with and without T2D. We used the measures of FPG variability including CV, SD, ARV, and VIM as a continuous variable (for each unit change) and tertiles in Cox PH models (the lowest tertile was considered as the reference). We also created four models in each dataset and adjusted for well known risk factors for CKD that were reported in a systematic review in this field. [40, 41]: Model 1: adjusted for age and sex at baseline (phase 2). Model 2: Model 1 + marital status, education, ever smoking, prevalent CVD, physical activity, anti-diabetic drug use, anti-hypertensive drug, lipid-lowering drug, BMI, WC, SBP, DBP, eGFR, and FPG at baseline (phase 2). Model 3: Model 1 + marital status, education, ever smoking, prevalent CVD, and physical activity at baseline (phase 2), and anti-diabetic drug use, anti-hypertensive drug, and lipid-lowering drug over phases 2-4, and average BMI, WC, SBP, DBP, and eGFR over phases 2. And Model 4: Model 3 + the average FPG during the measurement period. We conducted the above-mentioned analysis in the pooled data of TLGS and MESA cohorts using stratified Cox regression analysis and R version 3.6.2. Only, in the TLGS cohort, the above analysis was performed for 2-HPG among non-type 2 diabetic population; the association between this parameter with eGFR decline among newly diagnosed type 2 diabetic population was also examined only in Model 1. We calculated the median follow-up between 2002 (baseline phase) and 2018 (the end of the study) and assessed the PH assumptions in Cox models with the Schoenfeld residuals test and log-log plots, showing all proportional assumptions were appropriate. We performed the statistical analyses using STATA version 14. We also considered a P-value of <0.05 as statistically significant.

**Results**

***3.1. The TLGS cohort***

Participants included 528 patients with T2D (women = 326) with a mean (SD) age of 52.5 (10.6) years and 3894 participants without T2D (women = 2288) with a mean (SD) age of 40.5 (13.1) years. Baseline characteristics of the study population for participants with and without T2D across tertiles of FPG-SD are presented in **Table 1**. Generally, compared to the first tertile of FPG-SD, subjects with T2D at the third tertile had higher lipid-lowering drug use as well as higher FPG levels at baseline, phases 3, and 4. There was also a significant difference between ages of participants between teriles of FPG-SD. Moreover, among participants without T2D, those in the third tertile of FPG-SD generally had higher BMI, WC, and FPG levels at phases 3 and 4 compared to the reference group. There was also a significant difference between the levels of education of participants between teriles of FPG-SD.

After a median follow-up of 12.2 years (interquartile range: 11.1–13.3 years), 131 incident eGFR decline **≥** 30% and 72 incident eGFR decline **≥** 40% among subjects with T2D occurred; the corresponding values for non-diabetic participants were, 629 and 115, respectively.

**Table 2** shows the association of FPG variability as a continuous variable in four models with an eGFR decline **≥** 40%. Among participants without T2D, each unit change in FPG variability measures was significantly associated with a higher risk for eGFR decline **≥** 40% in all models of FPG-SD, FPG-CV, and FPG-VIM; the corresponding HRs and 95% CIs in the last models were 1.07 (1.01-1.13), 1.06 (1.01-1.11), and 1.07 (1.01-1.13), respectively. However, among participants with T2D, none of these measures were associated with eGFR decline **≥** 40% events, even in model 1.

The associations of FPG variability as a categorical variable with incident eGFR decline **≥** 40% are presented in **Table 3.** Generally, for SD and VIM in all of the models, a significant increasing trend was observed for incident eGFR decline among participants without T2D. Furthermore, among participants without T2D, the third tertiles of FPG-SD, and FPG-VIM were associated with higher risks in all models (the corresponding HRs and 95% CIs in the last model were 1.60 (1.01-2.54), and 1.69(1.06-2.69), respectively). Whereas, no trends for incident eGFR decline were observed in participants with T2D in any of the FPG variability measures.

**Supplementary Tables 1** and **2** show the association of FPG variability both as a continuous and categorical variable in four models with an eGFR decline **≥** 30%. Regardless of diabetes status, no association was shown between FPG variability measures and risk of eGFR decline in any of the models.

As a sensitivity analysis, we examined the association of 2-HPG variability both as continuous and categorical variables with eGFR declines **≥** 30% and 40% among participants without T2D and newly diagnosed T2D not on glucose lowering medications (**Supplementary Tables 3, 4**, **5, 6, and 7**, respectively). No association was shown between 2-HPG variability measures and risk of eGFR decline in any of the models in both non-diabetic individuals and newly diagnosed T2D participants.

***3.2. MESA cohort***

A population of 521 participants (women = 236) with T2D with a mean (SD) age of 63.2 (9.1) years and 3769 participants without T2D (women = 1968) with a mean (SD) age of 60.4 (9.9) years were evaluated. Baseline characteristics of the study population for participants with and without T2D across tertiles of FPG-SD are presented in **Table 4.** Compared to the first tertile of FPG-SD, subjects with T2D at the third tertile were generally younger, and had higher BMI as well as FPG levels at baseline, phases 2, and 3. Furthermore, among participants without T2D, those in the third tertile of FPG-SD (compared to the first tertile) were generally younger and had higher BMI, WC, and FPG levels in phases 3, and 4. There was also a significant difference between smoking status of participants between teriles of FPG-SD.

After a median follow-up of 9.2 years (interquartile range: 6.6–9.6 years), 106 incident eGFR decline **≥** 30% and 49 incident eGFR decline **≥** 40% among subjects with T2D occurred; the corresponding values for non-diabetic participants were, 157 and 62, respectively.

**Table 5** shows the association of FPG variability as a continuous variable in four models with an eGFR decline **≥** 40%. Among participants with T2D, each unit change in FPG variability measures was significantly associated with a higher risk for eGFR decline in model 4 of FPG-SD, FPG-CV, and FPG-VIM; the corresponding HRs and 95% CIs were 1.01 (1.00-1.02), 1.02 (1.00-1.03), and 1.01(1.00-1.02), respectively. However, among participants without T2D, none of these measures were associated with an eGFR decline **≥** 40%. The associations of FPG variability as a categorical variable with incident eGFR decline **≥** 40% are presented in **Table 6**. As shown in **Table 6**, a significant increasing trend was observed for incident eGFR decline among participants with T2D for FPG-SD in model 1, and FPG-ARV in both models 1 and 3. Furthermore, the third tertiles of FPG-SD (model 1), and FPG-ARV (models 1 and 3) were associated with significantly higher risks, the corresponding HRs and 95% CIs were 2.15 (1.03-4.50), 2.45 (1.16-5.16), and 2.31 (1.08-4.96), respectively. However, no trends were observed in participants without T2D.

The association between FPG variability measures and eGFR decline **≥** 30% was in line with the reported results for GFR decline **≥** 40%, except that one unit increase in FPG-ARV was significantly associated with a higher risk for eGFR decline in model 4 among T2D participants (1.01 (1.00-1.01)) (**Supplementary Tables 8** and **9**).

***3.3 Pooled data of TLGS and MESA cohorts***

The association between FPG variability measures and eGFR decline **≥** 40% as a continuous and categorical variable was also measured in pooled data of MESA and TLGS cohorts (**Tables 7 and 8,** respectively). Among participants without T2D, each unit change in FPG variability measures was significantly associated with a higher risk for eGFR decline in all models of FPG-SD, FPG-CV, and FPG-VIM; the corresponding HRs and 95% CIs of model 4 were 1.05 (1.01-1.10), 1.05 (1.00-1.09), and 1.05 (1.00-1.10), respectively. Among participants with T2D, only model 1 of FPG-ARV was associated with eGFR decline **≥** 40% (1.01(1.00-1.01)). However, no association was shown between FPG variability as a categorical variable and risk of eGFR decline in any of the models in both individuals with and without T2D in pooled data of TLGS and MESA cohorts. When we replaced eGFR decline ≥30% in place of eGFR ≥40% as the outcome of the study, no significant association was demonstrated for FPG variability measures in the fully adjusted model in both individuals and without T2D in the pooled data of two cohorts (data not shown)

**Discussion:**

For the first time, we examined the association between GV over 6 years assessed by SD, CV, ARV, and VIM and incident eGFR decline in both T2D and non-T2D individuals, separately in two well-known cohorts, namely TLGS and MESA during one a decade follow-up. Using eGFR decline ≥ 40% as the outcome, in the MESA, in the fully adjusted model, higher GV using SD and CV measures were significantly associated with a higher risk of eGFR decline among those with T2D; however, among those without T2D, no associations were found. In the TLGS, the higher GV using SD, CV, and VIM measures were significantly associated with a higher risk of eGFR decline only among those without T2D. Applying eGFR decline ≥ 30% as the outcome, in the TLGS, regardless of diabetes status, no association was shown between FPG variability measures and risk of eGFR decline; however, in the MESA the results were in line with those of GFR decline **≥**40%. Moreover, using pooled data from the two cohorts we found that each unit increase in FPG variability with all GV measurements excluding ARV were associated with about 5% higher risk of eGFR decline ≥40% only among non-T2D individuals.

To the best of our knowledge, several studies [42] have assessed the relationship between GV and incident CKD, ESRD, or diabetic kidney disease (DKD) and eGFR rate decline among T2D patients. GV was assessed by HbA1c variability in all of these studies except two studies [43, 44] that were assessed by both HbA1c and FPG variability.

Currently, eGFR decline is considered a validated surrogate endpoint for ESRD in randomized clinical trials (RCTs) as well as in cohort studies [25, 45, 46]. It was shown that while 20% and 30% eGFR decline are extremely susceptible to the existence of acute effects, 40% and 57% are more robust [26]. In an international meta-analysis of more than 1.7 million individuals with incident 12,344 ESRD events, it was shown that a decline in eGFR≥ 30% and ≥ 40% over a 3-year baseline period was associated with an adjusted HR of 7.0 (3.9-12.7) and 15.7 (7.4-33.4), respectively, compared to no changes in eGFR in those with baseline eGFR≥ 60 mL/min/1.73 m^2^ [25]. The corresponding values for T2D Japanese patients were estimated at 18.4 (7.6–44.7) and 12.8 (5.2–32.2) [45].

The following studies [31-33], assessed GV and eGFR decline among T2D patients. All of these studies evaluated the annual changes in eGFR among T2D individuals. Takenouchi et al. found that higher HbA1C-CV was associated with a higher risk of eGFR decline, mainly among those with an albumin/creatinine ratio ≥ 30 mg/g. In this study, no association was found between FPG -GV and eGFR decline [33]. Based on eGFR decline, Low et al. also found that renal disease progression was more common among those with T2D with higher HbA1C-CV independent of mean HbA1C. However, compared with patients with better average glycemic control, T2D patients with sub-optimal average glycemic control (i.e. HbA1C > 8.0%)were more likely to develop renal disease at lower magnitudes of HbA1C variability [32]. However, Lin Lee et al. found that even among T2D patients with well-controlled HbA1C levels (<7%), those with high HbA1C-CV still experienced faster eGFR decline[31]. In our data analysis, among T2D patients in MESA, with sub-optima mean FPG level about of 151 mg/dl, FPG variability had a greater likelihood for eGFR decline ≥ 30 and ≥ 40%.

To the best of our knowledge, there is also only one study that investigates the correlation between VVV and macrovascular and microvascular events in the general population [14]. In a 10-year prospective cohort study, Jang et al. found that the HbA1C-CV tertile was associated with an increased risk for macro-and microvascular events in non-DM middle-aged participants. The higher HbA1C variability was an independent risk factor for microvascular events, however for macrovascular events, the risk was more prominent for variabilities in FPG and post 2-hour blood glucose [14]. Our findings in MESA among non-T2D participants are consistent with this study, which found no associations between FPG variability measurements and eGFR decline ≥ 40%; however, FPG-SD, FPG-CV, and FPG-VIM were associated with higher eGFR decline ≥ 40% among non-T2D participants in TLGS.

Several studies have investigated the impact of ethnicity on eGFR decline [47], progression to ESRD, and development of CKD [48]. Peralta et al. assessed the ethnicity and racial disparities in kidney function decline in participants without CKD [47]. In age- and sex-adjusted models, black individuals had a higher risk of incident CKD among those with eGFR higher than 90 ml/min per 1.73 m^2^ as well as 60< eGFR≤ 90 ml/min per 1.73 m^2^, followed by Hispanics, while Chinese with 60< eGFR≤ 90 ml/min per 1.73 m2 had the lowest risk of incident CKD. The associations attenuated following adjustment for CKD risk factors, particularly hypertension and diabetes. Therefore, the authors concluded that the rate of kidney function decline before incident CKD could be different among various ethnicities, which cannot be fully explained by differences in CKD well-established risk factors [47]. The ethnicity disparity in CKD development in diabetic patients has also been revealed. Hull et al. found a higher rate of CKD development in South Asia relative to the White population [49]. Collectively, ethnicity can influence the rate of kidney function decline and its associations with different risk factors, which could be a reasonable explanation for our different association between GV and eGFR decline in the American versus Iranian cohort. Despite our best efforts, no previous studies addressed the impact of GV variability on eGFR decline in both T2D and non-T2D simultaneously.

From a pathophysiological perspective, although several potential mechanisms have been proposed to have the potential to connect enhanced GV with a higher risk of incident micro- and macro-vascular complications of diabetes including renal impairment, ESRD, and CKD, the exact mechanism has yet to be determined. There is a bulk of evidence [9, 50-52]  in support of the fact that short-term, as well as long-term GV can enhance inflammatory cytokines [53] , oxidative stress[53], and induce endothelial dysfunction[44, 54-56], all of which have been shown to have mandatory roles in diabetes complications. GV can increase human tubule-interstitial cell growth, collagen production, and endothelial apoptosis rates compared with persistent exposure to high glucose levels [55, 57, 58].

**Strengths and limitations**

The present study contains strengths that worth to be acknowledging. The primary strength of our study is that we examined the impact of exposure to GV on incident eGFR decline in two well-known cohorts among both participants with and without T2D during a long-term follow-up. Second, although the level of adjustment for potential risk factors is different among studies, we adjusted for well-known CKD risk factors in the current study.

On the other hand, several limitations should be mentioned. First, we did not have access to the data on HbA1C variability. Of note, Yang et al. demonstrated that both FPG-CV and HbA1c-CV can predict the development of ESRD in diabetes [44]. Furthermore, Jang et al. found out that while HbA1C variability is a better predictor of insulin resistance and inflammatory responses, FPG and 2-HPG are better predictors of sympathoadrenal activation, which was shown to be associated with hypoglycemia [14]. Second, we also did not have access to the data on the urine sample and the albuminuria status. Moreover, it was shown that eGFR is unreliable in detecting renal function in those with diabetes as it overestimates and underestimates measured GFR (mGFR) at lower mGFR and higher mGFR values, respectively [59]; thus, we used eGFR decline as a valid surrogate for renal failure similar to many RCTs [46] and few cohort studies [25, 46, 60]. Third, our study lacked data concerning the episodes of hypoglycemia, which was revealed to enhance the risk of CKD in those with T2D [61, 62]. Forth, we removed participants with eGFR decline ≥30% during the period of FPG fluctuations; however, since our exposure period did not proceed outcome in the strict sense (i.e. the time of start for eGFR decline and FPG variability assessment was the same), the absence of mentioned time lag may lead to inverse causality. Finally, the TLGS cohort was performed among residents of the metropolitan city of Tehran; thus, our findings cannot be extrapolated into rural zones of Iran and other ethnicities. It is important to note that the MESA cohort represents four different ethnicities including the white population which constitute the greatest part of the cohort (about 40%) followed by African American, Hispanic and Chinese Americans, aged ≥ 45 years [63], so it cannot be extrapolated to the younger age population.

**Conclusion:**

In summary, we found that higher FPG variability is associated with an increased risk of eGFR decline of ≥ 30 and ≥ 40% in the American population with diabetes. However, the unfavorable impact of FPG-GV was found only among the non-diabetic Iranian population for incident eGFR decline of ≥ 30%.

**Ethics approval**

The research protocol was approved by the ethics committee of the Research Institute for Endocrine Sciences, Shahid Beheshti University of Medical Sciences, Tehran, Iran. All methods were carried out by relevant guidelines and regulations. This study uses data from two cohort studies, and informed consent was obtained from all subjects and/or their legal guardian(s) not for this study directly, but during the data collection process in these two Iranian and American cohort studies.

**Availability of data and materials**

The data sets used and/or analyzed during the current study are available from the corresponding author upon reasonable request.

**Competing interests**

The authors have no financial involvement with any organization or any financial interest in or financial conflict with the subject or materials discussed in the manuscript. This manuscript has no relevant financial or other relationships to disclose. Peer reviewers of this manuscript have no relevant financial or other relationships to disclose.

**Funding**

None

**Authors’ contributions**

ND, YSH, and FH raised the presented idea and designed the study. FK and SM conducted the analyses. FH, YSH, ND, and FK interpreted the results. ND, YSH, FH, and SST developed the first draft of the manuscript. FH and FA critically reviewed the manuscript. All authors have read and approved the final manuscript.

**Consent of publication**

N/A

**Acknowledgments**

The authors would like to thank the participants and executive team of the TLGS for their passionate cooperation.

**References**

1. Brownlee M, Hirsch IB: **Glycemic variability: a hemoglobin A1c–independent risk factor for diabetic complications**. *Jama* 2006, **295**(14):1707-1708.

2. Lachin JM, Genuth S, Nathan DM, Zinman B, Rutledge BN, Group DER: **Effect of glycemic exposure on the risk of microvascular complications in the diabetes control and complications trial—revisited**. *Diabetes* 2008, **57**(4):995-1001.

3. Ayano-Takahara S, Ikeda K, Fujimoto S, Hamasaki A, Harashima S-i, Toyoda K, Fujita Y, Nagashima K, Tanaka D, Inagaki N: **Glycemic variability is associated with quality of life and treatment satisfaction in patients with type 1 diabetes**. *Diabetes Care* 2015, **38**(1):e1-e2.

4. DeVries JH: **Glucose variability: where it is important and how to measure it**. *Diabetes* 2013, **62**(5):1405.

5. Suh S, Kim JH: **Glycemic variability: how do we measure it and why is it important?** *Diabetes & metabolism journal* 2015, **39**(4):273-282.

6. Cavalot F: **Do data in the literature indicate that glycaemic variability is a clinical problem? Glycaemic variability and vascular complications of diabetes**. *Diabetes, Obesity and Metabolism* 2013, **15**(s2):3-8.

7. Smith-Palmer J, Brändle M, Trevisan R, Federici MO, Liabat S, Valentine W: **Assessment of the association between glycemic variability and diabetes-related complications in type 1 and type 2 diabetes**. *Diabetes research and clinical practice* 2014, **105**(3):273-284.

8. Jin SM, Kim TH, Oh S, Baek J, Joung J, Park SM, Cho Y, Sohn S, Hur K, Lee MS: **Association between the extent of urinary albumin excretion and glycaemic variability indices measured by continuous glucose monitoring**. *Diabetic Medicine* 2015, **32**(2):274-279.

9. Wadén J, Forsblom C, Thorn LM, Gordin D, Saraheimo M, Groop P-H, Group FDNS: **A1C variability predicts incident cardiovascular events, microalbuminuria, and overt diabetic nephropathy in patients with type 1 diabetes**. *Diabetes* 2009, **58**(11):2649-2655.

10. Muggeo M, Zoppini G, Bonora E, Brun E, Bonadonna RC, Moghetti P, Verlato G: **Fasting plasma glucose variability predicts 10-year survival of type 2 diabetic patients: the Verona Diabetes Study**. *Diabetes care* 2000, **23**(1):45-50.

11. Assessment G: **6. Glycemic Targets: Standards of Medical Care in Diabetes—2022**. *Diabetes Care* 2022, **45**:S83.

12. Committee ADAPP: **American Diabetes Association Professional Practice Committee:, Draznin B, et al. 11. Chronic Kidney Disease and Risk Management: Standards of Medical Care in Diabetes-2022**. *Diabetes Care* 2022, **45**:S175-S184.

13. Gorst C, Kwok CS, Aslam S, Buchan I, Kontopantelis E, Myint PK, Heatlie G, Loke Y, Rutter MK, Mamas MA: **Long-term Glycemic Variability and Risk of Adverse Outcomes: A Systematic Review and Meta-analysis**. *Diabetes Care* 2015, **38**(12):2354-2369.

14. Jang JY, Moon S, Cho S, Cho KH, Oh CM: **Visit-to-visit HbA1c and glucose variability and the risks of macrovascular and microvascular events in the general population**. *Sci Rep* 2019, **9**(1):1374.

15. Cardoso CRL, Leite NC, Moram CBM, Salles GF: **Long-term visit-to-visit glycemic variability as predictor of micro- and macrovascular complications in patients with type 2 diabetes: The Rio de Janeiro Type 2 Diabetes Cohort Study**. *Cardiovascular diabetology* 2018, **17**(1):33.

16. Barzegar N, Ramezankhani A, Tohidi M, Azizi F, Hadaegh F: **Long-term glucose variability and incident cardiovascular diseases and all-cause mortality events in subjects with and without diabetes: Tehran Lipid and Glucose Study**. *Diabetes Res Clin Pract* 2021, **178**:108942.

17. Penno G, Solini A, Bonora E, Fondelli C, Orsi E, Zerbini G, Morano S, Cavalot F, Lamacchia O, Laviola L *et al*: **HbA1c variability as an independent correlate of nephropathy, but not retinopathy, in patients with type 2 diabetes: the Renal Insufficiency And Cardiovascular Events (RIACE) Italian multicenter study**. *Diabetes Care* 2013, **36**(8):2301-2310.

18. Cummings DM, Larsen LC, Doherty L, Lea CS, Holbert D: **Glycemic control patterns and kidney disease progression among primary care patients with diabetes mellitus**. *J Am Board Fam Med* 2011, **24**(4):391-398.

19. Moazzeni SS, Arani RH, Hasheminia M, Tohidi M, Azizi F, Hadaegh F: **High Incidence of Chronic Kidney Disease among Iranian Diabetic Adults: Using CKD-EPI and MDRD Equations for Estimated Glomerular Filtration Rate**. *Diabetes & metabolism journal* 2021, **45**(5):684-697.

20. Feng XS, Farej R, Dean BB, Xia F, Gaiser A, Kong SX, Elliott J, Lindemann S, Singh R: **CKD Prevalence Among Patients With and Without Type 2 Diabetes: Regional Differences in the United States**. *Kidney Medicine* 2022, **4**(1):100385.

21. Bikbov B, Purcell CA, Levey AS, Smith M, Abdoli A, Abebe M, Adebayo OM, Afarideh M, Agarwal SK, Agudelo-Botero M: **Global, regional, and national burden of chronic kidney disease, 1990–2017: a systematic analysis for the Global Burden of Disease Study 2017**. *The lancet* 2020, **395**(10225):709-733.

22. Bouya S, Balouchi A, Rafiemanesh H, Hesaraki M: **Prevalence of Chronic Kidney Disease in Iranian General Population: A Meta-Analysis and Systematic Review**. *Ther Apher Dial* 2018, **22**(6):594-599.

23. Tohidi M, Hasheminia M, Mohebi R, Khalili D, Hosseinpanah F, Yazdani B, Nasiri AA, Azizi F, Hadaegh F: **Incidence of chronic kidney disease and its risk factors, results of over 10 year follow up in an Iranian cohort**. *PloS one* 2012, **7**(9):e45304-e45304.

24. Heerspink HJL, Tighiouart H, Sang Y, Ballew S, Mondal H, Matsushita K, Coresh J, Levey AS, Inker LA: **GFR decline and subsequent risk of established kidney outcomes: a meta-analysis of 37 randomized controlled trials**. *American journal of kidney diseases* 2014, **64**(6):860-866.

25. Coresh J, Turin TC, Matsushita K, Sang Y, Ballew SH, Appel LJ, Arima H, Chadban SJ, Cirillo M, Djurdjev O: **Decline in estimated glomerular filtration rate and subsequent risk of end-stage renal disease and mortality**. *Jama* 2014, **311**(24):2518-2531.

26. Greene T, Teng C-C, Inker LA, Redd A, Ying J, Woodward M, Coresh J, Levey AS: **Utility and validity of estimated GFR–based surrogate time-to-event end points in CKD: a simulation study**. *American journal of kidney diseases* 2014, **64**(6):867-879.

27. Inker LA, Heerspink HJL, Mondal H, Schmid CH, Tighiouart H, Noubary F, Coresh J, Greene T, Levey AS: **GFR decline as an alternative end point to kidney failure in clinical trials: a meta-analysis of treatment effects from 37 randomized trials**. *American journal of kidney diseases* 2014, **64**(6):848-859.

28. Levey AS, Inker LA, Matsushita K, Greene T, Willis K, Lewis E, De Zeeuw D, Cheung AK, Coresh J: **GFR decline as an end point for clinical trials in CKD: a scientific workshop sponsored by the National Kidney Foundation and the US Food and Drug Administration**. *American Journal of Kidney Diseases* 2014, **64**(6):821-835.

29. Badve SV, Palmer SC, Hawley CM, Pascoe EM, Strippoli GF, Johnson DW: **Glomerular filtration rate decline as a surrogate end point in kidney disease progression trials**. *Nephrology Dialysis Transplantation* 2016, **31**(9):1425-1436.

30. Chang WX, Asakawa S, Toyoki D, Nemoto Y, Morimoto C, Tamura Y, Ota T, Shibata S, Fujigaki Y, Shen ZY: **Predictors and the subsequent risk of end-stage renal disease–usefulness of 30% decline in estimated GFR over 2 years**. *PloS one* 2015, **10**(7):e0132927.

31. Lee CL, Chen CH, Wu MJ, Tsai SF: **The variability of glycated hemoglobin is associated with renal function decline in patients with type 2 diabetes**. *Ther Adv Chronic Dis* 2020, **11**:2040622319898370.

32. Low S, Lim SC, Yeoh LY, Liu YL, Liu JJ, Fun S, Su C, Zhang X, Subramaniam T, Sum CF: **Effect of long‐term glycemic variability on estimated glomerular filtration rate decline among patients with type 2 diabetes mellitus: Insights from the Diabetic Nephropathy Cohort in Singapore**. *Journal of Diabetes* 2017, **9**(10):908-919.

33. Takenouchi A, Tsuboi A, Terazawa-Watanabe M, Kurata M, Fukuo K, Kazumi T: **Direct association of visit-to-visit HbA1c variation with annual decline in estimated glomerular filtration rate in patients with type 2 diabetes**. *J Diabetes Metab Disord* 2015, **14**:69.

34. Azizi F, Zadeh-Vakili A, Takyar M: **Review of Rationale, Design, and Initial Findings: Tehran Lipid and Glucose Study**. *Int J Endocrinol Metab* 2018, **16**(4 Suppl):e84777.

35. Azizi F, Ghanbarian A, Momenan AA, Hadaegh F, Mirmiran P, Hedayati M, Mehrabi Y, Zahedi-Asl S: **Prevention of non-communicable disease in a population in nutrition transition: Tehran Lipid and Glucose Study phase II**. *Trials* 2009, **10**:5.

36. Bild DE, Bluemke DA, Burke GL, Detrano R, Diez Roux AV, Folsom AR, Greenland P, JacobsJr DR, Kronmal R, Liu K: **Multi-ethnic study of atherosclerosis: objectives and design**. *American journal of epidemiology* 2002, **156**(9):871-881.

37. Momenan AA, Delshad M, Sarbazi N, Rezaei Ghaleh N, Ghanbarian A, Azizi F: **Reliability and validity of the Modifiable Activity Questionnaire (MAQ) in an Iranian urban adult population**. *Arch Iran Med* 2012, **15**(5):279-282.

38. Jeon CY, Lokken RP, Hu FB, van Dam RM: **Physical activity of moderate intensity and risk of type 2 diabetes: a systematic review**. *Diabetes Care* 2007, **30**(3):744-752.

39. Mena L, Pintos S, Queipo NV, Aizpúrua JA, Maestre G, Sulbarán T: **A reliable index for the prognostic significance of blood pressure variability**. *J Hypertens* 2005, **23**(3):505-511.

40. Kazancioğlu R: **Risk factors for chronic kidney disease: an update**. *Kidney Int Suppl (2011)* 2013, **3**(4):368-371.

41. Habte-Asres HH, Wheeler DC, Forbes A: **The Association Between Glycaemic Variability and Progression of Chronic Kidney Disease: a Systematic Review**. *SN Comprehensive Clinical Medicine* 2022, **4**(1):102.

42. Habte-Asres HH, Wheeler DC, Forbes A: **The Association Between Glycaemic Variability and Progression of Chronic Kidney Disease: a Systematic Review**. *SN Comprehensive Clinical Medicine* 2022, **4**(1):1-14.

43. Lin C-C, Chen C-C, Chen F-N, Li C-I, Liu C-S, Lin W-Y, Yang S-Y, Lee C-C, Li T-C: **Risks of diabetic nephropathy with variation in hemoglobin A1c and fasting plasma glucose**. *The American journal of medicine* 2013, **126**(11):1017. e1011-1017. e1010.

44. Yang Y-F, Li T-C, Li C-I, Liu C-S, Lin W-Y, Yang S-Y, Chiang J-H, Huang C-C, Sung F-C, Lin C-C: **Visit-to-visit glucose variability predicts the development of end-stage renal disease in type 2 diabetes: 10-year follow-up of Taiwan diabetes study**. *Medicine* 2015, **94**(44).

45. Oshima M, Toyama T, Haneda M, Furuichi K, Babazono T, Yokoyama H, Iseki K, Araki S, Ninomiya T, Hara S: **Estimated glomerular filtration rate decline and risk of end-stage renal disease in type 2 diabetes**. *PloS one* 2018, **13**(8):e0201535.

46. Inker LA, Chaudhari J: **GFR slope as a surrogate endpoint for CKD progression in clinical trials**. *Current Opinion in Nephrology and Hypertension* 2020, **29**(6):581-590.

47. Peralta CA, Katz R, DeBoer I, Ix J, Sarnak M, Kramer H, Siscovick D, Shea S, Szklo M, Shlipak M: **Racial and ethnic differences in kidney function decline among persons without chronic kidney disease**. *Journal of the American Society of Nephrology* 2011, **22**(7):1327-1334.

48. Patzer RE, McClellan WM: **Influence of race, ethnicity and socioeconomic status on kidney disease**. *Nature Reviews Nephrology* 2012, **8**(9):533-541.

49. Dreyer G, Hull S, Mathur R, Chesser A, Yaqoob M: **Progression of chronic kidney disease in a multi‐ethnic community cohort of patients with diabetes mellitus**. *Diabetic medicine* 2013, **30**(8):956-963.

50. Ceriello A: **The possible role of postprandial hyperglycaemia in the pathogenesis of diabetic complications**. *Diabetologia* 2003, **46**(1):M9-M16.

51. Monnier L, Colette C, Rabasa-Lhoret R, Lapinski H, Caubel C, Avignon A, Boniface H: **Morning hyperglycemic excursions: a constant failure in the metabolic control of non–insulin-using patients with type 2 diabetes**. *Diabetes Care* 2002, **25**(4):737-741.

52. Sparkenbaugh E, Pawlinski R: **Interplay between coagulation and vascular inflammation in sickle cell disease**. *British Journal of Haematology* 2013, **162**(1):3-14.

53. Esposito K, Nappo F, Marfella R, Giugliano G, Giugliano F, Ciotola M, Quagliaro L, Ceriello A, Giugliano D: **Inflammatory cytokine concentrations are acutely increased by hyperglycemia in humans: role of oxidative stress**. *Circulation* 2002, **106**(16):2067-2072.

54. Ceriello A, Esposito K, Piconi L, Ihnat MA, Thorpe JE, Testa R, Boemi M, Giugliano D: **Oscillating Glucose Is More Deleterious to Endothelial Function and Oxidative Stress Than Mean Glucose in Normal and Type 2 Diabetic Patients**. *Diabetes* 2008, **57**(5):1349-1354.

55. Monnier L, Mas E, Ginet C, Michel F, Villon L, Cristol J-P, Colette C: **Activation of oxidative stress by acute glucose fluctuations compared with sustained chronic hyperglycemia in patients with type 2 diabetes**. *Jama* 2006, **295**(14):1681-1687.

56. Ceriello A, Ihnat M: **‘Glycaemic variability’: a new therapeutic challenge in diabetes and the critical care setting**. *Diabetic Medicine* 2010, **27**(8):862-867.

57. Brownlee M: **Biochemistry and molecular cell biology of diabetic complications**. *Nature* 2001, **414**(6865):813-820.

58. Brownlee M: **The pathobiology of diabetic complications: a unifying mechanism**. *diabetes* 2005, **54**(6):1615-1625.

59. Wood AJ, Churilov L, Perera N, Thomas D, Poon A, MacIsaac RJ, Jerums G, Ekinci EI: **Estimating glomerular filtration rate: performance of the CKD-EPI equation over time in patients with type 2 diabetes**. *Journal of Diabetes and its Complications* 2016, **30**(1):49-54.

60. Schrauben SJ, Shou H, Zhang X, Anderson AH, Bonventre JV, Chen J, Coca S, Furth SL, Greenberg JH, Gutierrez OM: **Association of multiple plasma biomarker concentrations with progression of prevalent diabetic kidney disease: findings from the chronic renal insufficiency cohort (CRIC) study**. *Journal of the American Society of Nephrology* 2021, **32**(1):115-126.

61. Yun J-S, Ko S-H, Ko S-H, Song K-H, Ahn Y-B, Yoon K-H, Park Y-M, Ko S-H: **Presence of macroalbuminuria predicts severe hypoglycemia in patients with type 2 diabetes: a 10-year follow-up study**. *Diabetes Care* 2013, **36**(5):1283-1289.

62. GERSTEIN H, MILLER M, BYINGTON R: **ACTION TO CONTROL CARDIOVASCULAR RISK IN DIABETES STUDY GROUP. EFFECTS OF INTENSIVE GLUCOSE LOWERING IN TYPE 2 DIABETES**. 2010.

63. Lakoski SG, Cushman M, Criqui M, Rundek T, Blumenthal RS, D'Agostino Jr RB, Herrington DM: **Gender and C-reactive protein: data from the Multiethnic Study of Atherosclerosis (MESA) cohort**. *American heart journal* 2006, **152**(3):593-598.

| Table 1. Baseline characteristics of participants across tertiles of SD for fasting plasma glucose in TLGS, Tehran Lipid and Glucose Study (2002–2018). | | | | | |
| --- | --- | --- | --- | --- | --- |
| Characteristics | With diabetes  (n=528) | T1  (n=176) | T2  (n=176) | T3  (n=176) | P value |
| Age (year) | **52.51 ± 10.64** | **52.37 ± 11.62** | **53.99 ± 9.84** | **51.18± 10.26** | **0.044** |
| Sex (women) | 326 (61.74) | 112 (63.64) | 97 (55.11) | 117 (66.48) | 0.074 |
| Marital status (married) | 457 (86.55) | 144 (81.82) | 156 (88.64) | 157 (89.20) | 0.078 |
| Education (high school and more) | 124 (23.48) | 42 (23.86) | 42 (23.86) | 40 (22.73) | 0.959 |
| Antihypertensive drug | 124 (23.48) | 48 (27.27) | 37 (21.02) | 39 (22.16) | 0.338 |
| Lipid-lowering drug | **81 (15.34)** | **18 (10.23)** | **25 (14.20)** | **38 (21.59)** | **0.011** |
| Current smoking | 109 (20.64) | 34 (19.32) | 43 (24.43) | 32 (18.18) | 0.304 |
| Moderate-high physical activity | 315 (59.66) | 103 (58.52) | 101 (57.39) | 111 (63.07) | 0.516 |
| BMI (Kg/m^2^) | 29.43 ± 4.75 | 29.61 ± 4.89 | 29.02 ± 4.30 | 29.65 ± 5.04 | 0.386 |
| WC (cm) | 98.87 ± 10.85 | 99.09 ± 11.22 | 98.67 ± 9.34 | 98.84 ± 11.39 | 0.937 |
| SBP (mmHg) | 127.91± 20.12 | 126.87± 19.61 | 129.55± 19.78 | 127.31± 20.96 | 0.408 |
| DBP (mmHg) | 78.35 ± 10.19 | 78.35 ± 10.19 | 79.87 ± 11.02 | 78.77 ± 10.50 | 0.380 |
| eGFR (mL/min/1.73 m2) | 88.67 ± 15.11 | 89.01± 16.06 | 87.82± 14.63 | 89.17 ± 14.63 | 0.659 |
| FPG (mg/dl) | **156.45 ± 53.00** | **132.38 ± 36.75** | **154.43± 40.29** | **182.55± 64.92** | **< 0.001** |
| FPG at phase 3 (mg/dl) | **130.66± 54.9** | **130.66 ± 37.70** | **150.39 ± 38.41** | **188.64 ± 68.55** | **< 0.001** |
| FPG at phase 4 (mg/dl) | **166.2 ± 60.6** | **135.39± 36.81** | **163.13± 42.28** | **198.53 ± 76.18** | **< 0.001** |
| Anti-diabetic drug at baseline | **231 (43.75)** | **60 (34.09)** | **75 (42.61)** | **96 (54.55)** | **0.001** |
|  | Without diabetes  (n=3,894) | T1  (n=1,331) | T2  (n=1,331) | T3  (n=1,331) | P value |
| Age (year) | 40.49 ± 13.09 | 40.18± 13.04 | 40.28 ± 13.02 | 41.02 ± 13.21 | 0.201 |
| Sex (women) | 2,288 (58.76) | 808 (60.71) | 740 (58.13) | 740 (57.36) | 0.190 |
| Marital status (married) | 3,144 (80.74) | 1,056 (79.34) | 1,027 (80.68) | 1,061 (82.25) | 0.168 |
| Education (high school and more) | **1,318 (33.85)** | **436 (32.76)** | **466 (36.61)** | **416 (32.25)** | **0.039** |
| Antihypertensive drug | 220 (5.65) | 79 (5.94) | 66 (5.18) | 75 (5.81) | 0.675 |
| Lipid-lowering drug | 113 (2.90) | 40 (3.01) | 26 (2.04) | 47 (3.64) | 0.052 |
| Current smoking | 812 (20.85) | 267 (20.06) | 258 (20.27) | 787 (61.01) | 0.318 |
| Moderate-high physical activity | 2458 (63.12) | 858 (64.46) | 813 (63.86) | 503 (38.99) | 0.149 |
| BMI (Kg/m^2^) | **26.82± 4.44** | **26.64 ± 4.44** | **26.76 ± 4.39** | **27.08 ± 4.49** | **0.033** |
| WC (cm) | **89.22 ± 11.70** | **88.34 ± 11.72** | **89.19 ± 11.40** | **90.15± 11.91** | **< 0.001** |
| SBP (mmHg) | 113.15 ± 16.00 | 112.36 ± 15.48 | 113.34 ± 16.16 | 113.79 ± 16.34 | 0.066 |
| DBP (mmHg) | 73.54 ± 10.07 | 73.11 ± 9.87 | 73.55± 10.25 | 73.98 ± 10.08 | 0.085 |
| eGFR (mL/min/1.73 m2) | 96.59 ± 16.57 | 96.77 ± 16.51 | 96.64 ± 16.22 | 96.37 ± 16.98 | 0.818 |
| FPG (mg/dl) | 88.44 ± 8.04 | 88.33 ± 6.75 | 88.63 ± 7.71 | 88.37 ± 9.47 | 0.579 |
| FPG at phase 3 (mg/dl) | **87.66 ± 7.74** | **88.11 ± 6.64** | **87.45 ± 7.48** | **87.41 ± 8.93** | **0.033** |
| FPG at phase 4 (mg/dl) | **93,20 ± 8.10** | **89.66 ± 6.51** | **92.53 ± 6.86** | **97.50 ± 8.73** | **< 0.001** |
| Data are represented as mean ± standard deviation for continuous variables and frequency (percent) for categorical variables.  T, tertile; CVD: cardiovascular disease; BMI: body mass index; WC, waist circumference; SBP: systolic blood pressure; DBP, diastolic blood pressure; eGFR: estimated glomerular filtration rate; FPG: fasting plasma glucose | | | | | |

| Table 2. HRs and 95% CIs of incident eGFR decline ≥ 40% according to each unit increase in FPG variability measures in Tehran Lipid and Glucose Study. | | | | | | | | | |
| --- | --- | --- | --- | --- | --- | --- | --- | --- | --- |
| Variability measures | | Model 1 | | Model 2 | | Model 3 | | Model 4 | |
|  |  | HR (95% CI) | P value | HR (95% CI) | P value | HR (95% CI) | P value | HR (95% CI) | P value |
| SD | |  |  |  |  |  |  |  |  |
|  | With diabetes | 1.00(0.99-1.01) | 0.645 | 0.99 (0.98-1.00) | 0.167 | 0.99 (0.99-1.00) | 0.258 | 0.99 (0.98-1.00) | 0.106 |
|  | Without diabetes | 1.08(1.02-1.13) | **0.006** | 1.06(1.01-1.12) | **0.020** | 1.07 (1.01-1.13) | **0.012** | 1.07 (1.01-1.13) | **0.014** |
| CV | |  |  |  |  |  |  |  |  |
|  | With diabetes | 0.99 (0.98-1.01) | 0.511 | 0.99 (0.97-1.01) | 0.158 | 0.99 (0.97-1.01) | 0.190 | 0.99 (0.97-1.00) | 0.138 |
|  | Without diabetes | 1.06(1.01-1.12) | **0.012** | 1.05 (1.00-1.11) | **0.033** | 1.06 (1.01-1.11) | **0.017** | 1.06 (1.01-1.11) | **0.017** |
| ARV | |  |  |  |  |  |  |  |  |
|  | With diabetes | 1.00(0.99-1.01) | 0.874 | 1.00 (0.99-1.00) | 0.310 | 1.00 (0.99-1.00) | 0.513 | 1.00 (0.99-1.00) | 0.309 |
|  | Without diabetes | 1.03(0.99-1.07) | 0.158 | 1.03(0.99-1.07) | 0.137 | 1.03 (0.99-1.07) | 0.183 | 1.03 (0.99-1.07) | 0.208 |
| VIM | |  |  |  |  |  |  |  |  |
|  | With diabetes | 1.00 (0.98-1.01) | 0.463 | 0.99 (0.98-1,00) | 0.252 | 0.99 (0.98-1.00) | 0.233 | 0.99 (0.98-1.00) | 0.250 |
|  | Without diabetes | 1.07(1.01-1.13) | **0.016** | 1.06 (1.00-1.11) | **0.041** | 1.07 (1.01-1.13) | **0.019** | 1.07 (1.01-1.13) | **0.018** |
| Model 1: adjusted for age and sex at baseline (phase 2)  Model 2: Model 1 + marital status, education, ever smoking, prevalent CVD, physical activity, anti-diabetic drug use, anti-hypertensive drug, lipid-lowering drug, BMI, WC, SBP, DBP, eGFR, and FPG at baseline (phase 2)  Model 3: Model 1 + marital status, education, ever smoking, prevalent CVD, and physical activity at baseline (phase 2), and anti-diabetic drug use, anti-hypertensive drug, and lipid-lowering drug over phases 2-4, and average BMI, WC, SBP, DBP, and eGFR over phases 2-4.  Model 4: Model 3 + average FPG | | | | | | | | | |

.

| Table 3. HRs and 95% CIs of incident eGFR decline ≥ 40% according to tertiles of FPG variability measures in Tehran Lipid and Glucose Study. | | | | |
| --- | --- | --- | --- | --- |
| Variability measures | Model 1 | Model 2 | Model 3 | Model 4 |
|  | HR (95% CI) | HR (95% CI) | HR (95% CI) | HR (95% CI) |
| With diabetes |  |  |  |  |
| SD |  |  |  |  |
| T1 | 1.00 (reference) | 1.00 (reference) | 1.00 (reference) | 1.00 (reference) |
| T2 | 1.20 (0.69-2.10) | 1.06 (0.59-1.90) | 0.93 (0.53-1.66) | 0.88 (0.49-1.58) |
| T3 | 0.98 (0.54-1.77) | 0.76 (0.39-1.47) | 0.77 (0.42-1.44) | 0.67 (0.33-1.33) |
| P _trend_ | 0.968 | 0.414 | 0.417 | 0.254 |
| CV |  |  |  |  |
| T1 | 1.00 (reference) | 1.00 (reference) | 1.00 (reference) | 1.00 (reference) |
| T2 | 1.04 (0.60-1.82) | 0.96 (0.53-1.73) | 0.80 (0.44-1.43) | 0.77 (0.42-1.39) |
| T3 | 0.94 (0.53-1.66) | 0.74 (0.40-1.37) | 0.74 (0.41-0.34) | 0.70 (0.38-1.29) |
| P _trend_ | 0.828 | 0.332 | 0.327 | 0.253 |
| ARV |  |  |  |  |
| T1 | 1.00 (reference) | 1.00 (reference) | 1.00 (reference) | 1.00 (reference) |
| T2 | 1.47 (0.84-2.58) | 1.29 (0.71-2.33) | 0.99 (0.55-1.79) | 0.94 (0.52-1.72) |
| T3 | 1.09 (0.60-2.01) | 0.84 (0.43-1.65) | 0.83 (0.44-1.55) | 0.71 (0.35-1.44) |
| P _trend_ | 0.749 | 0.588 | 0.539 | 0.344 |
| VIM |  |  |  |  |
| T1 | 1.00 (reference) | 1.00 (reference) | 1.00 (reference) | 1.00 (reference) |
| T2 | 1.10 (0.63-1.92) | 1.18 (0.67-2.09) | 1.09 (0.62-1.92) | 1.10 (0.62-1.93) |
| T3 | 0.94 (0.53-1.66) | 0.84 (0.46-1.51) | 0.82 (0.46-1.47) | 0.83 (0.46-1.49) |
| P _trend_ | 0.835 | 0.577 | 0.512 | 0.746 |
| Without diabetes |  |  |  |  |
| SD |  |  |  |  |
| T1 | 1.00 (reference) | 1.00 (reference) | 1.00 (reference) | 1.00 (reference) |
| T2 | 1.28 (0.80-2.07) | 1.37 (0.84-2.21) | 1.32 (0.81-2.13) | 1.31 (0.81-2.12) |
| T3 | **1.61 (1.02-2.53)** | **1.67 (1.05-2.64)** | **1.61 (1.02-2.55)** | **1.60 (1.01-2.54)** |
| P _trend_ | **0.039** | **0.029** | **0.041** | **0.047** |
| CV |  |  |  |  |
| T1 | 1.00 (reference) | 1.00 (reference) | 1.00 (reference) | 1.00 (reference) |
| T2 | 1.36 (0.85-2.18) | 1.43 (0.89-2.30) | 1.43 (0.90-2.31) | 1.44 (0.89-2.30) |
| T3 | 1.53 (0.97-2.42) | 1.56 (0.98-2.48) | 1.55 (0.98-2.47) | 1.55 (0.97-2.46) |
| P _trend_ | 0.071 | 0.064 | 0.065 | 0.068 |
| ARV |  |  |  |  |
| T1 | 1.00 (reference) | 1.00 (reference) | 1.00 (reference) | 1.00 (reference) |
| T2 | 1.08 (0.68-1.70) | 1.18 (0.74-1.88) | 1.21 (0.76-1.93) | 1.21 (0.76-1.92) |
| T3 | 1.27 (0.82-1.96) | 1.38 (0.89-2.15) | 1.30 (0.84-2.01) | 1.28 (0.82-1.99) |
| P _trend_ | 0.286 | 0.147 | 0.242 | 0.272 |
| VIM |  |  |  |  |
| T1 | 1.00 (reference) | 1.00 (reference) | 1.00 (reference) | 1.00 (reference) |
| T2 | 1.42 (0.88-2.28) | 1.49 (0.93-2.41) | 1.49 (0.93- 2.39) | 1.49 (0.92-2.40) |
| T3 | **1.65 (1.04-2.62)** | **1.66 (1.04-2.67)** | **1.69 (1.06-2.69)** | **1.69(1.06-2.69)** |
| P _trend_ | **0.034** | **0.035** | **0.029** | **0.029** |
| Model 1: adjusted for age and sex at baseline (phase 2)  Model 2: Model 1 + marital status, education, ever smoking, prevalent CVD, physical activity, anti-diabetic drug use, anti-hypertensive drug, lipid-lowering drug, BMI, WC, SBP, DBP, eGFR, and FPG at baseline (phase 2)  Model 3: Model 1 + marital status, education, ever smoking, prevalent CVD, and physical activity at baseline (phase 2), and anti-diabetic drug use, anti-hypertensive drug, and lipid-lowering drug over phases 2-4, and average BMI, WC, SBP, DBP, and eGFR over phases 2-4.  Model 4: Model 3 + average FPG | | | | |

| Table 4. Baseline characteristics of the participants across tertiles of SD for fasting plasma glucose in Multi-Ethnic Study of Atherosclerosis study. | | | | | |
| --- | --- | --- | --- | --- | --- |
| Characteristics | With diabetes  (n=521) | T1  (n=176) | T2  (n=172) | T3  (n=173) | P value |
| Age (year) | 63.21 ± 9.13 | 64.85 ± 8.82 | 64.67 ± 8.85 | 60.10 ± 8.96 | **< 0.001** |
| Sex (women) | 236 (45.30) | 83 (47.16) | 72 (41.86) | 81 (46.82) | 0.541 |
| Marital status (married) | 320 (61.42) | 108 (61.36) | 110 (63.95) | 102 (58.96) | 0.635 |
| Education (high school and more) | 281 (53.93) | 105 (59.66) | 86 (50.00) | 90 (52.02) | 0.161 |
| Antihypertensive drug | 317 (60.84) | 106 (60.23) | 114 (66.28) | 97 (56.07) | 0.148 |
| Lipid-lowering drug | 142 (27.26) | 47 (26.70) | 47 (27.33) | 48 (27.75) | 0.976 |
| Current smoking | 259 (49.71) | 82 (46.59) | 88 (51.16) | 89 (51.45) | 0.595 |
| Moderate-high physical activity | 279 (53.55) | 96 (54.55) | 91 (52.51) | 92 (53.18) | 0.947 |
| BMI (Kg/m^2^) | **30.51 ± 5.63** | **29.59 ± 5.18** | **30.88 ± 6.05** | **31.08 ± 5.58** | **0.028** |
| WC (cm) | 104.67 ± 14.19 | 102.84 ± 13.17 | 105.77 ± 15.06 | 105.43 ± 14.20 | 0.107 |
| SBP (mmHg) | 130.45 ± 20.19 | 129.15 ± 20.94 | 133.31 ± 19.96 | 128.95 ± 19.44 | 0.077 |
| DBP (mmHg) | 71.70 ± 9.39 | 70.91 ± 10.15 | 71.91 ± 8.65 | 72.29 ± 9.29 | 0.366 |
| eGFR (mL/min/1.73 m2) | 91.86 ± 20.19 | 88.19 ± 16.99 | 89.37 ± 19.02 | 98.06 ± 22.79 | **< 0.001** |
| FPG (mg/dl) | **150.22 ± 53.21** | **127.13 ± 31.31** | **142.42 ± 39.19** | **181.46 ± 66.68** | **< 0.001** |
| FPG at phase 3 (mg/dl) | **153.43 ± 58.79** | **126.86 ± 30.96** | **141.61 ± 36.31** | **192.23 ± 76.08** | **< 0.001** |
| FPG at phase 4 (mg/dl) | **141.00 ± 50.74** | **127.35 ± 30.08** | **137.37 ± 39.12** | **158.49 ± 69.49** | **< 0.001** |
| Anti-diabetic drug at baseline | 390 (74.86) | 128 (72.73) | 134 (77.91) | 128 (73.99) | 0.511 |
|  | Without diabetes  (n=3,769) | T1  (n=1,278) | T2  (n=1,253) | T3  (n=1,238) | P value |
| Age (year) | **60.41 ± 9.89** | **60.78 ± 9.83** | **60.62 ± 9.91** | **59.83 ± 9.90** | **0.036** |
| Sex (women) | 1,968 (52.22) | 685 (53.60) | 631 (50.36) | 652 (52.67) | 0.245 |
| Marital status (married) | 2,394 (63.52) | 804 (62.91) | 815 (65.04) | 775 (62.60) | 0.385 |
| Education (high school and more) | 2,630 (69.78) | 885 (69.25) | 873 (69.67) | 872 (70.44) | 0.806 |
| Antihypertensive drug | 1,059 (28.10) | 349 (27.31) | 338 (26.98) | 372 (30.05) | 0.173 |
| Lipid-lowering drug | 490 (13.00) | 154 (12.05) | 159 (12.69) | 177 (14.30) | 0.227 |
| Current smoking | **1,858 (49.30)** | **591 (46.24)** | **642 (51.24)** | **625 (50.48)** | **0.025** |
| Moderate-high physical activity | 2321 (61.58) | 797 (62.36) | 763 (60.89) | 761 (61.47) | 0.746 |
| BMI (Kg/m^2^) | **27.57 ± 4.99** | **27.08 ± 4.80** | **27.40 ± 4.85** | **28.24 ± 5.25** | **< 0.001** |
| WC (cm) | **95.77 ± 13.52** | **94.56 ± 12.95** | **95.45 ± 13.27** | **97.34 ± 14.18** | **< 0.001** |
| SBP (mmHg) | 122.92 ± 20.21 | 123.13 ± 20.37 | 122.65 ± 19.95 | 122.97 ± 20.31 | 0.834 |
| DBP (mmHg) | 71.55 ± 10.03 | 71.63 ± 10.00 | 71.25 ± 9.84 | 71.79 ± 10.24 | 0.388 |
| eGFR (mL/min/1.73 m2) | 82.29 ± 13.76 | 82.31 ± 13.56 | 82.14 ± 13.40 | 82.42 ± 14.34 | 0.875 |
| FPG (mg/dl) | 87.51 ± 8.90 | 88.80 ± 7.45 | 87.67 ± 8.38 | 87.06 ± 10.63 | 0.089 |
| FPG at phase 3 (mg/dl) | **90.64 ± 8.58** | **88.56 ± 7.17** | **89.88 ± 7.63** | **93.57 ± 9.83** | **< 0.001** |
| FPG at phase 4 (mg/dl) | **90.22 ± 8.71** | **88.39 ± 7.33** | **89.81 ± 8.18** | **92.52 ± 9.96** | **< 0.001** |
| Data are represented as mean ± standard deviation for continuous variables and frequency (percent) for categorical variables.  T, tertile; CVD: cardiovascular disease; BMI: body mass index; WC, waist circumference; SBP: systolic blood pressure; DBP, diastolic blood pressure; eGFR: estimated glomerular filtration rate; FPG: fasting plasma glucose | | | | | |

| Table 5. HRs and 95% CIs of incident eGFR decline ≥ 40% according to each unit increase in FPG variability measures in Multi-Ethnic Study of Atherosclerosis. | | | | | | | | | |
| --- | --- | --- | --- | --- | --- | --- | --- | --- | --- |
| Variability measures | | Model 1 | | Model 2 | | Model 3 | | Model 4 | |
|  |  | HR (95% CI) | P value | HR (95% CI) | P value | HR (95% CI) | P value | HR (95% CI) | P value |
| SD | |  |  |  |  |  |  |  |  |
|  | With diabetes | 1.01(1.01-1.02) | **0.001** | 1.01(1.00-1.02) | **0.040** | 1.01(1.01-1.02) | **0.002** | 1.01 (1.00-1.02) | **0.042** |
|  | Without diabetes | 1.03(0.94-1.12) | 0.516 | 1.02(0.94-1.11) | 0.615 | 1.01(0.93-1.10) | 0.762 | 1.01(0.93-1.10) | 0.790 |
| CV | |  |  |  |  |  |  |  |  |
|  | With diabetes | 1.02 (1.01-1.04) | **0.008** | 1.02(1.00-1.03) | 0.067 | 1.02(1.00-1.03) | **0.020** | 1.02 (1.00-1.03) | **0.041** |
|  | Without diabetes | 1.02(0.94-1.10) | 0.635 | 1.02(0.94-1.10) | 0.631 | 1.01(0. 93-1.09) | 0.797 | 1.01(0.93-1.09) | 0.799 |
| ARV | |  |  |  |  |  |  |  |  |
|  | With diabetes | 1.01(1.00-1.01) | **0.006** | 1.01(1.00-1.01) | **0.026** | 1.01(1.00-1.01) | **0.007** | 1.01 (1.00-1.01) | 0.087 |
|  | Without diabetes | 1.00(0.94-1.07) | 0.889 | 1.00(0.94-1.07) | 0.990 | 0.99(0.93-1.06) | 0.827 | 0.99(0.93-1.06) | 0.794 |
| VIM | |  |  |  |  |  |  |  |  |
|  | With diabetes | 1.01(1.00-1.02) | 0.052 | 1.00(1.01-1.02) | 0.119 | 1.01(1.00-1.02) | 0.144 | 1.01(1.00-1.02) | 0.055 |
|  | Without diabetes | 1.02(0.93-1.11) | 0.656 | 1.02(0.93-1.12) | 0.633 | 1.01(0.93-1.10) | 0.803 | 1.01(0.93-1.10) | 0.799 |
| Model 1: adjusted for age and sex at baseline (phase 2)  Model 2: Model 1 + marital status, education, ever smoking, prevalent CVD, physical activity, anti-diabetic drug use, anti-hypertensive drug, lipid-lowering drug, BMI, WC, SBP, DBP, eGFR, and FPG at baseline (phase 2)  Model 3: Model 1 + marital status, education, ever smoking, prevalent CVD, and physical activity at baseline (phase 2), and anti-diabetic drug use, anti-hypertensive drug, and lipid-lowering drug over phases 2-4, and average BMI, WC, SBP, DBP, and eGFR over phases 2-4  Model 4: Model 3 + average FPG | | | | | | | | | |

.

| Table 6. HRs and 95% CIs of incident eGFR decline ≥ 40% according to tertiles of FPG variability measures in Multi-Ethnic Study of Atherosclerosis. | | | | |
| --- | --- | --- | --- | --- |
| Variability measures | Model 1 | Model 2 | Model 3 | Model 4 |
|  | HR (95% CI) | HR (95% CI) | HR (95% CI) | HR (95% CI) |
| With diabetes |  |  |  |  |
| SD |  |  |  |  |
| T1 | 1.00 (reference) | 1.00 (reference) | 1.00 (reference) | 1.00 (reference) |
| T2 | 1.48 (0.69-3.19) | 1.28 (0.58-2.81) | 1.26 (0.57-2.77) | 1.18 (0.53-2.60) |
| T3 | **2.15 (1.03-4.50)** | 1.38 (0.59-2.22) | 1.96 (0.93-4.17) | 1.46 (0.64-3.34) |
| P _trend_ | **0.040** | 0.459 | 0.069 | 0.367 |
| CV |  |  |  |  |
| T1 | 1.00 (reference) | 1.00 (reference) | 1.00 (reference) | 1.00 (reference) |
| T2 | 0.81 (0.37-1.79) | 0.65 (0.29-1.49) | 0.70 (0.31-1.57) | 0.66 (0.29-1.49) |
| T3 | 1.78 (0.91-3.47) | 1.32 (0.65-2.70) | 1.60 (0.81-3.16) | 1.35 (0.67-2.75) |
| P _trend_ | 0.073 | 0.326 | 0.128 | 0.307 |
| ARV |  |  |  |  |
| T1 | 1.00 (reference) | 1.00 (reference) | 1.00 (reference) | 1.00 (reference) |
| T2 | 1.48(0.67-3.30) | 1.16 (0.50-2.68) | 1.35 (0.60-3.06) | 1.20 (0.53-2.75) |
| T3 | **2.45 (1.16-5.16)** | 1.79 (0.79-4.08) | **2.31 (1.08-4.96)** | 1.78 (0.78-4.06) |
| P _trend_ | **0.015** | 0.132 | **0.025** | 0.145 |
| VIM |  |  |  |  |
| T1 | 1.00 (reference) | 1.00 (reference) | 1.00 (reference) | 1.00 (reference) |
| T2 | 0.54 (0.24-1.23) | 0.51 (0.22-1.18) | 0.50 (0.21-1.13) | 0.48 (0.21-1.12) |
| T3 | 1.52 (0.80-2.87) | 1.39 (0.72-2.69) | 1.35 (0.70-2.61) | 1.49 (0.76-2.93) |
| P _trend_ | 0.155 | 0.234 | 0.264 | 0.178 |
| Without diabetes |  |  |  |  |
| SD |  |  |  |  |
| T1 | 1.00 (reference) | 1.00 (reference) | 1.00 (reference) | 1.00 (reference) |
| T2 | 0.57 (0.30-1.09) | 0.59 (0.31-1.14) | 0.59(0.31-1.13) | 0.59 (0.30-1.13) |
| T3 | 0.93 (0.53-1.64) | 0.95 (0.53-1.68) | 0.88 (0.49-1.56) | 0.87 (0.49-1.55) |
| P _trend_ | 0.744 | 0.804 | 0.623 | 0.603 |
| CV |  |  |  |  |
| T1 | 1.00 (reference) | 1.00 (reference) | 1.00 (reference) | 1.00 (reference) |
| T2 | 0.74 (0.40-1.36) | 0.79 (0.43-1.45) | 0.76 (0.41-1.40) | 0.76 (0.41-1.40) |
| T3 | 0.82 (0.45-1.49) | 0.86 (0.47-1.58) | 0.80 (0.44-1.46) | 0.80 (0.44-1.46) |
| P _trend_ | 0.483 | 0.600 | 0.445 | 0.444 |
| ARV |  |  |  |  |
| T1 | 1.00 (reference) | 1.00 (reference) | 1.00 (reference) | 1.00 (reference) |
| T2 | 0.77 (0.42-1.39) | 0.74 (0.41-1.35) | 0.74 (0.41-1.35) | 0.74 (0.41-1.34) |
| T3 | 0.70 (0.38-1.30) | 0.69 (0.37-1.28) | 0.65 (0.35-1.21) | 0.64 (0.34-1.19) |
| P _trend_ | 0.243 | 0.215 | 0.160 | 0.148 |
| VIM |  |  |  |  |
| T1 | 1.00 (reference) | 1.00 (reference) | 1.00 (reference) | 1.00 (reference) |
| T2 | 0.67 (0.36-1.24) | 0.73 (0.39-1.36) | 0.70 (0.38-1.30) | 0.70 (0.38-1.29) |
| T3 | 0.78 (0.43-1.41) | 0.83 (0.45-1.52) | 0.77 (0.43-1.40) | 0.77 (0.43-1.41) |
| P _trend_ | 0.384 | 0.516 | 0.372 | 0.375 |
| Model 1: adjusted for age and sex at baseline (phase 2)  Model 2: Model 1 + marital status, education, ever smoking, prevalent CVD, physical activity, anti-diabetic drug use, anti-hypertensive drug, lipid-lowering drug, BMI, WC, SBP, DBP, eGFR, and FPG at baseline (phase 2)  Model 3: Model 1 + marital status, education, ever smoking, prevalent CVD, and physical activity at baseline (phase 2), and anti-diabetic drug use, anti-hypertensive drug, and lipid-lowering drug over phases 2-4, and average BMI, WC, SBP, DBP, and eGFR over phases 2-4  Model 4: Model 3 + average FPG | | | | |

| Table 7. HRs and 95% CIs of incident eGFR decline ≥ 40% according to each unit increase in FPG variability measures in pooled data of Tehran Lipid and Glucose Study and Multi-Ethnic Study of Atherosclerosis cohorts. | | | | | | | | | |
| --- | --- | --- | --- | --- | --- | --- | --- | --- | --- |
| Variability measures | | Model 1 | | Model 2 | | Model 3 | | Model 4 | |
|  |  | HR (95% CI) | P value | HR (95% CI) | P value | HR (95% CI) | P value | HR (95% CI) | P value |
| SD | |  |  |  |  |  |  |  |  |
|  | With diabetes | 1.01(1.00-1.01) | 0.054 | 1.00 (0.99-1.01) | 0.767 | 1.00 (1.00-1.01) | 0.175 | 1.00 (0.99-1.01) | 0.881 |
|  | Without diabetes | **1.06(1.01-1.11)** | **0.010** | **1.05(1.01-1.10)** | **0.025** | **1.05 (1.01-1.10)** | **0.024** | **1.05 (1.01-1.10)** | **0.025** |
| CV | |  |  |  |  |  |  |  |  |
|  | With diabetes | 1.01 (1.00-1.02) | 0.147 | 1.00 (0.99-1.01) | 0.799 | 1.00 (0.99-1.02) | 0.394 | 1.00 (0.99-1.02) | 0.725 |
|  | Without diabetes | **1.05(1.01-1.09)** | **0.020** | **1.04 (1.00-1.09)** | **0.038** | **1.05 (1.00-1.09)** | **0.030** | **1.05 (1.00-1.09)** | **0.030** |
| ARV | |  |  |  |  |  |  |  |  |
|  | With diabetes | **1.01(1.00-1.01)** | **0.026** | 1.00 (1.00-1.01) | 0.411 | 1.00 (1.00-1.01) | 0.105 | 1.00 (1.00-1.01) | 0.590 |
|  | Without diabetes | 1.02(0.99-1.06) | 0.248 | 1.02 (1.09-1.06) | 0.231 | 1.02 (0.98-1.05) | 0.323 | 1.02 (0.98-1.05) | 0.343 |
| VIM | |  |  |  |  |  |  |  |  |
|  | With diabetes | 1.00 (1.00-1.01) | 0.438 | 1.00 (0.99-1.01) | 0.806 | 1.00 (0.99-1.01) | 0.825 | 1.00 (0.99-1.01) | 0.580 |
|  | Without diabetes | **1.05(1.01-1.10)** | **0.025** | **1.05 (1.00-1.10)** | **0.043** | **1.05 (1.00-1.10)** | **0.032** | **1.05 (1.00-1.10)** | **0.032** |
| Model 1: adjusted for age and sex at baseline (phase 2)  Model 2: Model 1 + marital status, education, ever smoking, prevalent CVD, physical activity, anti-diabetic drug use, anti-hypertensive drug, lipid-lowering drug, BMI, WC, SBP, DBP, eGFR, and FPG at baseline (phase 2)  Model 3: Model 1 + marital status, education, ever smoking, prevalent CVD, and physical activity at baseline (phase 2), and anti-diabetic drug use, anti-hypertensive drug, and lipid-lowering drug over phases 2-4, and average BMI, WC, SBP, DBP, and eGFR over phases 2-4.  Model 4: Model 3 + average FPG | | | | | | | | | |

| Table 8. HRs and 95% CIs of incident eGFR decline ≥ 40 % according to tertiles of FPG variability measures in pooled data of Tehran Lipid and Glucose Study and Multi-Ethnic Study of Atherosclerosis cohorts. | | | | |
| --- | --- | --- | --- | --- |
| Variability measures | Model 1 | Model 2 | Model 3 | Model 4 |
|  | HR (95% CI) | HR (95% CI) | HR (95% CI) | HR (95% CI) |
| With diabetes |  |  |  |  |
| SD |  |  |  |  |
| T1 | 1.00 (reference) | 1.00 (reference) | 1.00 (reference) | 1.00 (reference) |
| T2 | 1.29 (0.82-2.02) | 1.05 (0.66-1.66) | 1.08 (0.69-1.71) | 1.00 (0.63-1.59) |
| T3 | 1.41 (0.89-2.21) | 0.92 (0.56-1.52) | 1.19 (0.75-1.90) | 0.92 (0.55-1.56) |
| P _trend_ | 0.141 | 0.734 | 0.462 | 0.766 |
| CV |  |  |  |  |
| T1 | 1.00 (reference) | 1.00 (reference) | 1.00 (reference) | 1.00 (reference) |
| T2 | 0.95 (0.60-1.49) | 0.75 (0.47-1.19) | 0.80 (0.50-1.26) | 0.74 (0.47-1.18) |
| T3 | 1.27 (0.83-1.95) | 0.92 (0.59-1.44) | 1.07 (0.69-1.65) | 0.93 (0.59-1.47) |
| P _trend_ | 0.268 | 0.788 | 0.730 | 0.837 |
| ARV |  |  |  |  |
| T1 | 1.00 (reference) | 1.00 (reference) | 1.00 (reference) | 1.00 (reference) |
| T2 | 1.45 (0.92-2.29) | 1.09 (0.68-1.76) | 1.13 (0.71-1.81) | 1.05 (0.65-1.69) |
| T3 | 1.58 (0.99-2.49) | 1.08 (0.65-1.79) | 1.31 (0.82-2.11) | 1.04 (0.62-1.76) |
| P _trend_ | 0.055 | 0.781 | 0.253 | 0.885 |
| VIM |  |  |  |  |
| T1 | 1.00 (reference) | 1.00 (reference) | 1.00 (reference) | 1.00 (reference) |
| T2 | 0.87 (0.55-1.37) | 0.84 (0.53-1.33) | 0.83 (0.52-1.31) | 0.84 (0.53-1.33) |
| T3 | 1.19 (0.78-1.81) | 1.06 (0.69-1.62) | 1.05 (0.68-1.62) | 1.11 (0.72-1.71) |
| P _trend_ | 0.423 | 0.779 | 0.805 | 0.629 |
| Without diabetes |  |  |  |  |
| SD |  |  |  |  |
| T1 | 1.00 (reference) | 1.00 (reference) | 1.00 (reference) | 1.00 (reference) |
| T2 | 0.94 (0.65-1.37) | 0.99 (0.68-1.45) | 0.96 (0.66-1.41) | 0.96 (0.66-1.41) |
| T3 | 1.28 (0.90-1.82) | 1.32 (0.93-1.88) | 1.26 (0.88-1.79) | 1.25 (0.88-1.79) |
| P _trend_ | 0.161 | 0.123 | 0.197 | 0.208 |
| CV |  |  |  |  |
| T1 | 1.00 (reference) | 1.00 (reference) | 1.00 (reference) | 1.00 (reference) |
| T2 | 1.07 (0.74-1.54) | 1.13 (0.78-1.63) | 1.12 (0.77-1.62) | 1.12 (0.77-1.62) |
| T3 | 1.20 (0.84-1.73) | 1.25 (0.87-1.79) | 1.20 (0.84-1.73) | 1.20 (0.84-1.72) |
| P _trend_ | 0.310 | 0.235 | 0.320 | 0.324 |
| ARV |  |  |  |  |
| T1 | 1.00 (reference) | 1.00 (reference) | 1.00 (reference) | 1.00 (reference) |
| T2 | 0.94 (0.65-1.34) | 0.99 (0.69;1.42) | 0.99 (0.69-1.42) | 0.98 (0.68-1.42) |
| T3 | 1.02 (0.72-1.45) | 1.06 (0.75-1.51) | 1.02 (0.71-1.45) | 1.01 (0.71-1.44) |
| P _trend_ | 0.918 | 0.743 | 0.938 | 0.973 |
| VIM |  |  |  |  |
| T1 | 1.00 (reference) | 1.00 (reference) | 1.00 (reference) | 1.00 (reference) |
| T2 | 1.06 (0.73-1.53) | 1.12 (0.78-1.63) | 1.11 (0.77-1.61) | 1.11 (0.77-1.61) |
| T3 | 1.24 (0.87-1.78) | 1.28 (0.89-1.84) | 1.25 (0.87-1.79) | 1.25 (0.87-1.80) |
| P _trend_ | 0.234 | 0.184 | 0.224 | 0.223 |
| Model 1: adjusted for age and sex at baseline (phase 2)  Model 2: Model 1 + marital status, education, ever smoking, prevalent CVD, physical activity, anti-diabetic drug use, anti-hypertensive drug, lipid-lowering drug, BMI, WC, SBP, DBP, eGFR, and FPG at baseline (phase 2)  Model 3: Model 1 + marital status, education, ever smoking, prevalent CVD, and physical activity at baseline (phase 2), and anti-diabetic drug use, anti-hypertensive drug, and lipid-lowering drug over phases 2-4, and average BMI, WC, SBP, DBP, and eGFR over phases 2-4.  Model 4: Model 3 + average FPG | | | | |

.

**Figure 1 caption: TLGS study participants’ flowchart.**

**Figure 2 caption: MESA study participants’ flowchart.**

**
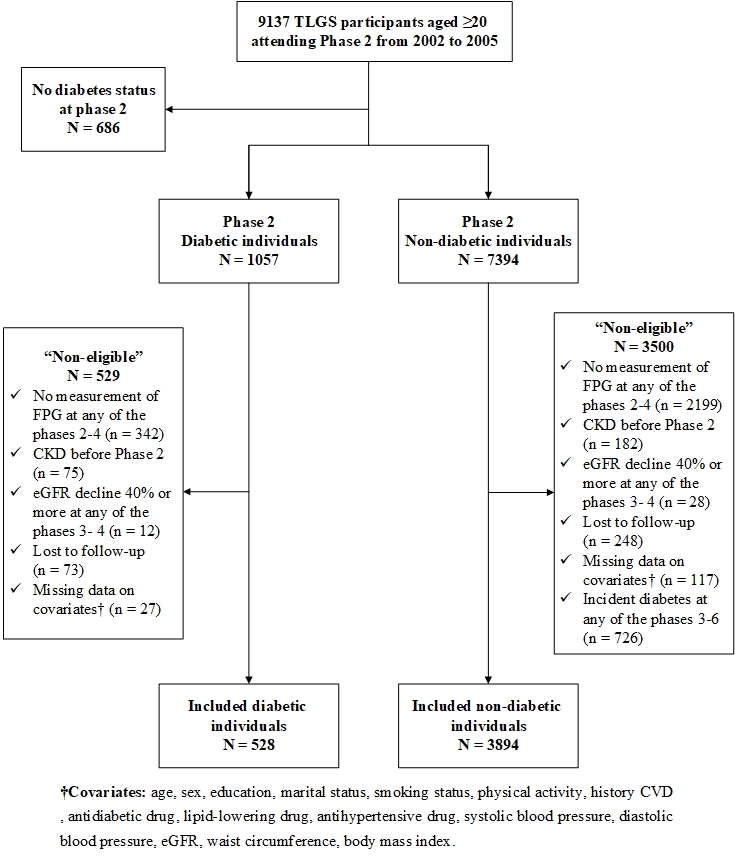
Figure 1: TLGS study participants’ flowchart.**


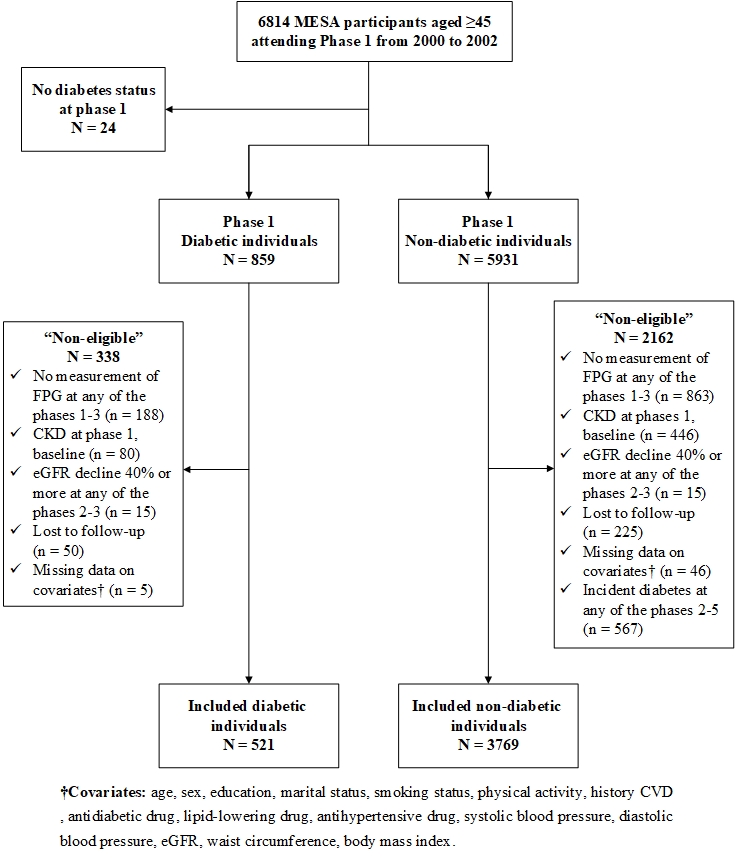
 **Figure 2: MESA study participants’ flowchart.**
